# Proteinortho6: pseudo-reciprocal best alignment heuristic for graph-based detection of (co-)orthologs

**DOI:** 10.3389/fbinf.2023.1322477

**Published:** 2023-12-13

**Authors:** Paul Klemm, Peter F. Stadler, Marcus Lechner

**Affiliations:** ^1^ Center for Synthetic Microbiology (SYNMIKRO), Philipps-Universität Marburg, Marburg, Germany; ^2^ Bioinformatics Group, Institute of Computer Science and Interdisciplinary Center for Bioinformatics, Leipzig University, Leipzig, Germany; ^3^ Max-Planck-Institute for Mathematics in the Sciences, Leipzig, Germany; ^4^ Institute for Theoretical Chemistry, University of Vienna, Wien, Austria; ^5^ Facultad de Ciencias, Universidad National de Colombia, Bogotá, Colombia; ^6^ Santa Fe Institute, Santa Fe, NM, United States

**Keywords:** orthology, homology, sequence similarity, spectral clustering, algebraic connectivity

## Abstract

Proteinortho is a widely used tool to predict (co)-orthologous groups of genes for any set of species. It finds application in comparative and functional genomics, phylogenomics, and evolutionary reconstructions. With a rapidly increasing number of available genomes, the demand for large-scale predictions is also growing. In this contribution, we evaluate and implement major algorithmic improvements that significantly enhance the speed of the analysis without reducing precision. Graph-based detection of (co-)orthologs is typically based on a reciprocal best alignment heuristic that requires an all vs. all comparison of proteins from all species under study. The initial identification of similar proteins is accelerated by introducing an alternative search tool along with a revised search strategy—the pseudo-reciprocal best alignment heuristic—that reduces the number of required sequence comparisons by one-half. The clustering algorithm was reworked to efficiently decompose very large clusters and accelerate processing. Proteinortho6 reduces the overall processing time by an order of magnitude compared to its predecessor while maintaining its small memory footprint and good predictive quality.

## 1 Introduction

Comparative analyses of nucleic and amino acid sequences have become routine approaches in modern biology. A problem frequently encountered in comparative and functional genomics as well as in phylogenomics and evolutionary reconstructions is the detection of homologous genes that share an evolutionary ancestry. These genes are orthologs if they have derived from a common ancestor by means of a speciation event. Paralogs, in contrast, have derived from a duplication event and thus represent gene copies (Fitch, 1970). Orthologs are of particular interest as their function is likely conserved due to selective pressure (ortholog conjecture (Koonin, 2005)). In contrast, paralogs diverge faster, specialize, acquire new functions, or become dysfunctional (Ohno, 1999; Lynch and Conery, 2000). Gene duplications followed by subsequent speciation events create two or more genes in one lineage that are, collectively, orthologous to one or more genes in another lineage. These sets of genes are termed co-orthologs (Koonin, 2005). Even though orthology is not a transitive relation (Johnson, 2007), large-scale orthology assessment is often treated as a clustering problem, resulting in *clusters of (co)-orthologous genes* (COGs), see, e.g., (Setubal and Stadler, 2018) for a review. Proteinortho (Lechner et al., 2011) in its previous version 5 (Proteinortho5) is a well-established tool for the detection of (co-)orthologs in large-scale analysis that also adheres to this approach. It has demonstrated its utility in various studies within the field of comparative genomics including, e.g., evolutionary analyses (Peter et al., 2018), genomic signatures (Kapheim et al., 2015), functional annotation (Pinho et al., 2013), phylogenetic reconstructions (Klemm et al., 2022), and so on. Proteinortho also found integration into tools and databases, such as Echinobase (Arshinoff et al., 2022) or Funannotate (Palmer and Stajich, 2023).

Sequence-based orthology inference is based on pairwise sequence comparisons. This stage requires scoring the similarity of all proteins in order to determine groups with high similarity. To simplify the terminology, we use the term “protein” to designate the amino acid representation of protein-coding gene sequences in the following. The well-known reciprocal best alignment heuristic (RBAH) (Bork et al., 1998), can be used to retrieve at least a good approximation of the correct ortholog set. We refer to Schaller et al. (2021) for a comprehensive mathematical analysis of the relation between best matches and orthology. Proteinortho extends the RBAH to an adaptive version, which includes alternative matches to the set of potential orthologs if they closely resemble the similarity of the best match. For details, refer to the original implementation (Lechner et al., 2011). Pairwise sequence comparisons are typically the most time-consuming stage as the computational effort scales quadratically to the number of proteomes analyzed.

When all pairwise sets of reciprocal best hits are known, this information is merged. In this process, all proteins are represented as nodes in a graph that are connected by edges whenever their similarity score is within the adaptive RBAH criterion. A set of proteins linked to each other by any path is called a connected component (CC). Each CC represents a potential co-orthologous group. However, the small world phenomenon (Milgram, 1967) also applies to empirical orthology graphs: Even though the number of possible protein sequences is practically limitless, there are relatively few basic folding shapes, of which some folds and superfamilies are extremely abundant (Koonin et al., 2002). CCs quickly become large and thereby non-informative. This effect increases with the number of proteins analyzed at once. Therefore, a clustering step is required. CCs are divided into smaller, more informative CCs by iteratively isolating well-connected subsets. The results are clusters of mutually similar proteins reported as co-orthologous groups.

In this contribution, we evaluate major algorithmic improvements for Proteinortho and present version 6 of the tool (Proteinortho6). All improvements primarily aim towards a significant speedup of orthology analyses while keeping the quality of its results and the small memory footprint that makes it applicable on large HPC systems and average off-the-shelf desktop systems.

## 2 Methods

### 2.1 Alternative sequence search tools

The first stage of Proteinortho analyses is a pairwise sequence comparison. Proteinortho5 relies on BLAST (Camacho et al., 2009) which is still considered the gold standard for any homology search (Ward and Moreno-Hagelsieb, 2014). BLAST implements a seed-and-extend paradigm. Meanwhile, it has inspired numerous alternative algorithms that can be used as direct replacements. Here, we evaluate these alternatives for use in the context of the adaptive RBAH strategy in order to speed up the sequence comparisons performed for orthology inference.

Proteinortho6 directly supports the following BLAST alternatives: ucsc BLAT is optimized for quickly finding very similar sequences of closely related species. It uses an index of non-overlapping k-mers (Kent, 2002) to speed up the search. UBLAST uses spaced seeds and a reduced alphabet to facilitate the comparison of distant gene sequences with a low identity (Edgar, 2010). USEARCH instead requires exact matches and was designed for comparisons of sequences with a high identity (Edgar, 2010). LAST implements a suffix array for a variable seed length, spaced seeds, and a reduced alphabet. A design goal was to handle repeat-rich sequences more efficiently than other tools (Kiełbasa et al., 2011). The parameter m (default 10) controls the maximum initial matches per query position comparable to the max_target_seqs parameter of BLAST. The higher the m, the more hits are reported at the cost of increased running time and memory usage. RAPSearch2 is based on a collision-free hash table of sorted 6-mers and a reduced alphabet for amino acid sequences (Zhao et al., 2012). DIAMOND implements a double index alignment, spaced seeds, and a reduced database alphabet (Buchfink et al., 2015). It provides several sensitivity modes depending on the expected sequence identity of reported hits. The default is optimized for hits >60% identity and short-read alignment. The fast mode aims for highly similar hits with >90% . The sensitive mode is recommended for comparisons above >40% sequence identity, while the highest sensitivity setting ultra-sensitive is supposed to perform well even below 40% identity, although with largely increased running time. MMSeqs2 uses a memory-efficient inexact *k*-mer matching optimized for multi-core systems (Steinegger and Söding, 2017). Speed and sensitivity can be controlled with the s parameter. A reasonable range starts from 1, corresponding with fast but coarse results, to 7.5, which is highly sensitive but slow. The default value is 5.7 and thus aims towards sensitivity over speed. Topaz is the most recent addition of BLAST replacements. It uses an advanced version of the SANS algorithm (Koskinen and Holm, 2012) that generalizes the symmetric suffix array neighborhood search to an asymmetric search in combination with scored seeds, a variation of spaced seeds (Medlar and Holm, 2018). Similarly to the tools above, a fast mode is implemented that decreases running time at the expense of sensitivity.

The results obtained using BLAST were considered as the point of reference. Based on these, we computed sensitivity and precision, where sensitivity = TP/(TP+FN) and precision = TP/(TP+FP) and TP is the number of true positive reported edges, that coincide with BLAST, FP is the number of false-positive reported edges, that do not coincide with BLAST, and FN is the number of false-negatives edges, that are only reported by BLAST. Computational efficiency was quantified in terms of total running time (wall time), scalability (running time in relation to the number of species), and maximal memory allocation (peak memory consumption). The evaluation was performed using the following tool versions: BLAST+ (v2.13.0), ucsc BLAT (v377), UBLAST and USEARCH (v11.0.667), LAST (v1318), RAPSearch2 (v2.24), DIAMOND (v2.0.15), MMSeqs2 (v14.7e284), and topaz (commit 24bdb61).

Note that the free 32-bit versions of USEARCH and UBLAST were used instead of the 64-bit versions that are available only commercially. Even though these versions are likely faster, we do not expect that the sensitivity and precision of the tool are affected by the build architecture.

### 2.2 Pseudo-reciprocal sequence comparison strategy

Pairwise similarity scores between all proteins in the dataset are the foundation of sequence-based orthology inference via adaptive RBAH. For reasons of complexity, only scores below a certain expectation value (E-value) are considered. In Proteinortho, sets of proteins *S*_1_, *S*_2_, … , *S*_*n*_ are presented for each species of interest. Similarity scores are then calculated using a sequence search tool *st*, like BLAST. This is performed reciprocally for all pairs of sets, e.g., *st*(*S*_1_, *S*_2_), *st*(*S*_2_, *S*_1_), *st*(*S*_1_, *S*_3_), *st*(*S*_3_, *S*_1_), ⋯ in order to obtain all scores required for RBAH. Notably, the alignments of any two proteins *a* ∈ *S*_*n*_ and *b* ∈ *S*_*m*_ are calculated twice if their match is below the E-value threshold in the comparisons *st*(*S*_*n*_, *S*_*m*_) and *st*(*S*_*m*_, *S*_*n*_).

The new feature pseudo (pseudo-reciprocal) in Proteinortho6, calculates only one pair *st*(*S*_*n*_, *S*_*m*_) and approximates the results of the *st*(*S*_*m*_, *S*_*n*_). The missing E-values of *st*(*S*_*m*_, *S*_*n*_) are calculated based on the query sequence length *l*, and the database size |*S*_*n*_| of the respective set of proteins in order to resemble E-values comparable to a pair-wise search:

$$e=\frac{l\cdot |S_{n}|}{2^{\mathrm{bitscore}}}$$

### 2.3 Clustering algorithm

#### 2.3.1 Eigenvector decomposition

Proteinortho uses a spectral clustering algorithm. It recursively divides connected components into two connected subcomponents that are maximally connected with respect to their algebraic connectivity (Fiedler, 1975). Spectral clustering has a long history in multivariate statistics, image processing, and machine learning, see, e.g., Shi and Malik (2000) for detailed descriptions. The implementation is based on the eigenvector decomposition of subgraphs, which are calculated via the power iteration in Proteinortho5 (Boutsidis et al., 2015). As large components usually build up due to bridge and hub clusters, most nodes within a connected component are not connected by an edge which is exploited by representing the data via a space-efficient edge list rather than a largely unoccupied adjacency matrix. This data structure is also well utilized by the power iteration. In contrast to alternative implementations based on adjacency matrices, non-existing edges do not require memory nor do they require consideration during the calculations. The strategy enables large-scale clustering by minimizing memory requirements and computational effort (Lechner et al., 2011).

In addition to the power iteration, Proteinortho6 implements ssyevr (single precision, symmetric eigenvalue problem, RRR algorithm). It is based on the “Relatively Robust Representation” algorithm (Parlett and Dhillon, 2000) that can compute an eigenpair in linear time (Bientinesi et al., 2005) which is provided via the highly optimized Fortran 77 library Lapack (v3.8.0) (Anderson et al., 1999). Although ssyevr outperforms the power iteration by orders of magnitude in many scenarios, the Lapack routine cannot be applied for large clusters of protein as it is bound by quadratic memory requirements due to the reliance on adjacency matrices.

#### 2.3.2 Flooding heuristic

With a growing number of species that are analyzed at once, connected components in orthology graphs grow exponentially in size due to the small world phenomenon. The resulting CCs can quickly cover a large proportion of the whole protein set. An example of this observation is shown in Supplementary Data Sheet S1. Theoretically, these huge CCs are easily broken down into informative subsets by spectral clustering. However, with an increasing number of species, their size poses a computational problem. The power iteration algorithm is not able to process them in a reasonable time while the memory requirements for ssyevr are not feasible. Hence, orthologs in these large CCs cannot be recovered.

To salvage the issue with large CCs, Proteinortho6 employs an iterative approach that removes batches of outlier edges based on their associated bitscore when spectral clustering is not possible. Therefore a cutoff threshold is raised until a significant number of outliers is covered with respect to the one-sided Grubb-Smirnov outlier test. If necessary, this process is repeated until spectral clustering is possible.

#### 2.3.3 Multithreading

Proteinortho6 introduces support for parallel computing at the clustering stage. The main thread employs a breadth-first search (BFS) approach to identify CCs. The worker threads then calculate the algebraic connectivity in parallel for each CC. Split components are added back to the processing queue if necessary. This feature also facilitates distribution across multiple computing nodes by processing batches of connected components in parallel. An overview can be found in Supplementary Data Sheet S1.

#### 2.3.4 Adaptive clustering

The spectral clustering approach follows a bisecting paradigm. Groups are successively divided until a predefined algebraic connectivity threshold is met. The choice of this threshold directly affects the size and quality of reported (co-)orthologous groups. A high connectivity threshold will only return sets of mutually similar proteins but can lead to excessive fragmentation of the orthology graph in numerous small CCs. Orthologous groups might fall apart into several subsets. A low threshold, on the other hand, might return non-informative large CCs with multiple putative co-orthologs for each species that actually represent unions of several orthologous groups. The default threshold applied by Proteinortho was defined empirically and represents a reasonable trade-off between both extremes.

Different protein families have different overall similarities. Therefore, a connectivity threshold that works well for one protein family, might be suboptimal for another. To address this, Proteinortho6 offers an adaptive clustering with the core option. It assumes that members of orthologous groups should be found in all species. Iterative spectral clustering is applied irrespective of the graph’s connectivity until the graph would split into two subgraphs of which neither covers all species that were covered by the original CC. The CC is only clustered further if it appears too big, e.g., comprises many (co-)orthologous genes per species. This threshold is defined by the parameter coreMaxProts (default 10), which continues clustering if more than 10 proteins are present per species.

### 2.4 Evaluation

#### 2.4.1 Datasets

Several real-world datasets were used as a biologically relevant basis for representative comparisons. These are summarized in Supplementary Table S1. It shows the number of species and proteins for each dataset and how this translates into a reciprocal best hit graph (RBH) using Proteinortho6 with BLAST (E-Value threshold 10^–5^).

The dataset QfO_2020/04_ was provided by the QfO benchmark service (Altenhoff et al., 2016). It comprises a curated set of proteomes from 23 Bacteria, 7 Archaea, and 48 Eukaryota sampled from UniProt (UniProt-Consortium, 2018). Note that QfO provides two versions of this dataset, and we used the newer version with updated UP000008143 sequences.

The Bac dataset comprised all bacterial reference proteomes from UniProt, release 2022/03 (UniProt-Consortium, 2018). This set was downsampled to incremental subsets of random proteomes. For instance, Bac_10_ contains 10 randomly selected bacterial proteomes, Bac_20_ extends this set by 10 additionally randomly selected proteomes, and so on. A full list is shown in Supplementary Table S1.

The BigCC set comprises connected components of 1,800 bacteria for which an origin of replication was identified (related study not published so far). Due to a huge connected component, this dataset represents a challenge for the clustering algorithm. So far, it was not solvable using regular spectral clustering. A subset of this is BigCC100 which focuses on larger CCs with at least 100 nodes. To evaluate edge cases that were not covered by this real-world dataset, such as components with high density and a large number of nodes, a set of 300 simulated graphs was generated. The set will be referred to as simulated. Its connected components were generated in three steps: An unweighted path graph was generated with the given number of nodes *n* and *n* − 1 edges connecting each node in a series to ensure connectivity. Edges were added one by one, randomly assigning unconnected nodes until the given graph density was satisfied. Bitscores were defined randomly (between 1 and 2000). E-Values were trivially set to 1/bitscore .

#### 2.4.2 Benchmark system

All benchmarks were conducted on the HPC cluster MaRC3 located at the University of Marburg using AMD EPYC 7702P processors with 64 cores and 256 GB RAM.

#### 2.4.3 Clustering algorithms

The spectral clustering algorithms were applied to the datasets BigCC100 and simulated, representing particularly large connected components. A total of 8,881 connected components were evaluated in this way, see Supplementary Table S1. If the relative clustering time differed by less than 5 min or one log_2_ fold, the algorithms were considered to be equally fast. To evaluate the comparability of both clustering approaches, the adjusted rand index (ARI) was used (Hubert and Arabie, 1985). The higher the ARI value, the more similar the partitioning.

#### 2.4.4 Precision of orthology predictions

The QfO benchmark service was used to evaluate the orthology predictions (Altenhoff et al., 2016). The Nextflow implementation of the benchmark system was used as provided in the corresponding GitHub repository Altenhoff (2023). All benchmarks were performed using the QfO_2020/04_ (2020.2) dataset. In this analysis, the precision metrics of the three categories of benchmarks were employed.

1. Phylogeny-based benchmarks GSTD2 (4 tests), the generalized species tree discordance, as well as the STD (3 tests), the species tree discordance, using the Average Robinson-Foulds (RF) distance between predicted gene trees based on the set of orthologs and the underlying species tree (the lower the better). The RF metric is a dissimilarity measure that quantifies the difference between two trees by counting the number of partitions that can be observed in one phylogenetic tree but not the other and *vice versa*. This metric can be seen as an approximation of the false discovery rate or the inverse of precision Altenhoff et al. (2016).
2. Function-based benchmarks used EC, the Enzyme Classification Conservation, and GO, the Gene Ontology Conservation, through the Average Schlicker Similarity as a proxy for precision (the higher, the better). The Average Schlicker Similarity is a semantic similarity measure used to assess the terms.
3. Reference Orthology-based benchmarks examined the agreement with the SwissTree, VGNC or TreeFam-A gene phylogeny, measured by the Positive Predictive Value (PPV, the higher, the better).

Full details on the test statistics can be found in (Altenhoff et al., 2016).

To combine the precision metrics of the different benchmarks, we define improvement as the mean log_2_ fold ratio between all scores. The scores of Proteinortho v5.16b with default settings serve as the baseline and, for example, an improvement of 0.5 corresponds to scores that are on average 41% better than the results of Proteinortho5.

#### 2.4.5 Scalability

The evaluation was performed with the Bac datasets of increasing size (Bac_10_, Bac_20_, ⋯). The following tools were evaluated in the comparison: OrthoFinder v2.5.4 (Emms and Kelly, 2019) in the graph-based modus (-og) using MMSeqs2 v14.7e284, SonicParanoid2 v1.3.8 (Cosentino and Iwasaki, 2023) using DIAMOND v2.0.15 in sensitive mode, OMA v2.5.0 (Altenhoff et al., 2019) without an out-group set, Proteinortho5 v5.16b utilizing BLAST v2.13.0, and Proteinortho6 v6.3.0 with DIAMOND v2.0.15 in sensitive mode, as well as the pseudo-reciprocal variation. A full list of all dependencies, versions, and parameters is provided in Supplementary Table S1.

## 3 Results

### 3.1 Sequence search tools

Pairwise similarity data is fundamental to graph-based orthology inference. The computation of all vs. all comparisons using a sequence search tool is also the most costly step. Typically, BLAST was the search tool of choice. It offers excellent performance compared to directly calculating scores from pairwise alignments and is considered the gold standard in terms of sensitivity and precision (Ward and Moreno-Hagelsieb, 2014). To our knowledge, more modern search tools are less accurate in general but perform much better in respect to processing time and memory consumption (see Table 1). We systematically compared potential alternatives to BLAST in the context of Proteinortho’s adaptive reciprocal best-hit heuristic (Lechner et al., 2011). The evaluation is based on the QfO 2020/04 dataset (Altenhoff et al., 2016), which comprises a representative mix of eukaryotic, bacterial, and archaeal proteomes.

**TABLE 1:** Performance and resource consumption of sequence search tools in the context of Proteinortho based on the QfO benchmark dataset 2020/04. Alternative search modes are listed below the tool’s names. The default option is indicated (def.). Sensitivity and precision are given relative to the BLAST results in line 1. Edges: number of edges in the initial orthology graph; wall time: total processing time; memory: peak memory usage; l_2_FC: log_2_ fold change relative to Proteinortho5 results; *: default option of Proteinortho6, pseudo: pseudo-reciprocal sequence comparison strategy as described in 2.2. Ranks are indicated: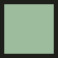 top 25%,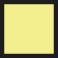 top 50%.

Algorithm	Edges	Sensitivity	Precision	Wall time		Memory	
		%	%	l_2_FC	*h*	l_2_FC	GB
Proteinortho5	5435 k	100	100	0	77.8	0	97
DIAMOND							
default	4701 k	77	89	7.2	0.5	4.1	6
sensitive	5366 k	88.4	89.5	6.4	0.9	4	6
sensitive + pseudo*	5417 k	88.7	88.9	7.5	0.4	4.3	5
ultrasens	5457 k	89.7	89.3	4.6	3.2	3.8	7
fast	3894 k	63.8	89	7.3	0.5	4.4	4
LAST							
m10 (def.)	4853 k	79.5	89	6.6	0.8	3.5	9
m100	5118 k	84.2	89.4	5.1	2.3	2.3	20
m1000	5239 k	86.2	89.5	2.2	16.5	1.9	25
MMSeqs2							
s1	3877 k	64	89.7	6.9	0.7	3.4	9
s5.7 (def.)	5149 k	85.6	90.4	4.5	3.5	2.9	13
s7.5	5235 k	87.1	90.5	2.7	12	2.9	13
topaz							
default	5025 k	82.3	89	4	4.9	3.2	10
fast	5025 k	82.3	89	4.1	4.5	3.2	11
USEARCH							
ublast	5167 k	81.1	85.3	5.5	1.8	2.1	23
usearch	3215 k	51.8	87.5	7.6	0.4	5.5	2
ucsc BLAT	1158 k	20	94	7.8	0.3	5.5	2
RAPSearch2	2781 k	46.6	91.1	2.2	16.9	3.1	11

The original implementation of Proteinortho5 relies on BLAST. It required 97 GB of memory and about 3 days (78 h) of processing time in total. Table 1 shows that both running time as well as memory consumption improve significantly if alternative search tools are used. In terms of precision, ucsc BLAT stands out with 94% and is best in total processing time (21 min, 7.8 log_2_ fold improvement) as well as memory footprint (2 GB, 5.5 log_2_ fold improvement over BLAST). However, this tool returns the lowest number of edges and achieves the by far worst sensitivity of all options (20%). Similarly, RAPSearch2, and USEARCH also fall behind in terms of sensitivity (47% and 52%, respectively). The remaining tools are close regarding precision (around 90%) and sensitivity (usually between 80% and 90%). With respect to both measures of quality, DIAMOND, LAST, MMSeqs2, topaz, and UBLAST could serve as suitable BLAST replacements when applying the right search mode. Factoring in processing time and memory requirements, DIAMOND with the sensitive option was evaluated to be the most optimal approach.

Using DIAMOND with the sensitive option as the search tool improved the running time by a log_2_ fold of 6.4 (to 56 min instead of 77.8 h) and the memory consumption by 4 log_2_ units (peak memory usage of 6 GB instead of 97 GB). In addition, we applied the pseudo-reciprocal sequence comparison strategy, pseudo. Here, protein alignments are calculated only in one direction while the reverse direction is estimated. See the Methods section for details. This approach additionally speeds up the calculation by half. Compared to the classic search strategy, the measures of quality are hardly affected. Precision decreases from 89.5% to 88.9% while sensitivity increases from 88.4% to 88.7%.

Comparable outcomes were noted for a group of closely related species and for randomly selected bacterial proteomes from the Bac_*n*_ dataset. For additional details, please refer to Supplementary Data Sheet S1. The pseudo-reciprocal best alignment heuristic using DIAMOND with the sensitive option, therefore, became the new default for Proteinortho6.

### 3.2 Clustering algorithm

Once pairwise similarity data was merged into an overarching graph structure, spectral clustering is applied to reduce it to an orthology graph. Proteinortho recursively divides connected components into two connected subcomponents that are maximally connected with respect to their algebraic connectivity. For this process, the space-efficient power iteration is used in Proteinortho5. With Proteinortho6, the ssyevr algorithm is available as an alternative. It relies on full matrices and is thus less space-efficient. We conducted a comprehensive evaluation of the running time differences between the power iteration and ssyevr algorithms using the real-world dataset BigCC100 was used together with a simulated set that comprises components with high density and a large number of nodes. See the Methods section for details.

Lapacks ssyevr has a significantly larger memory footprint for large connected components. The maximal requirement for processing a CC in the reference datasets was around 18 MB (ssyevr) vs. 0.1 MB (power iteration), see Supplementary Table S1 for details. Given the availability of system memory in modern computer systems, these additional requirements are largely outweighed by the improvement in performance. The maximal relative improvement in running time was 9.3 log_2_ folds for a graph with 1,921 nodes (4 s using ssyevr vs. 40 min the power iteration), and the maximum absolute running time difference was 1.36 h for a simulated graph with 7,731 nodes. 217 out of the 8,881 connected components were processed significantly faster using ssyevr over the power iteration. The improvement was 5.1 log_2_ folds on average. Our evaluation shows that the ssyevr implementation is consistently faster for large components and on par with the power iteration for small components. For this reason, the power iteration was replaced by the ssyevr as the default clustering algorithm in Proteinortho6.

Notably, the chosen algorithm scales quadratically in memory with the number of nodes. The BigCC100 dataset already comprises a connected component with 4 million nodes which exceed feasible computing capacities. While the power iteration would be able to handle this component from a memory perspective, the processing time would largely exceed any reasonable value. We stopped the comparative evaluation of clustering this component after 10 days. The increasing appearance of large connected components with an increase of species that are analyzed for (co-)orthologous proteins is expected due to the small world phenomenon (Milgram, 1967). We found a number of additional components in real-world datasets that are close in size. Hence, a large proportion of the proteins cannot be assigned to any (co-)orthologous group, if the components are ignored. To avoid a loss of information due to this effect, Proteinortho6 employs a flooding heuristic. Low-scoring edges are iteratively removed from large components until they are decomposed to sufficiently small subcomponents that are suitable for spectral clustering. See Methods sections for details.

### 3.3 Pseudo-reciprocal best alignment heuristic

To assess the validity of the pseudo approach, the reciprocal best hit graph from the QfO 2020/04 data sets was evaluated using the classic RBAH and the pseudo approach. Here, bitscores calculated by DIAMOND differ by 1.1% in median and 1.9% on average for any pairs of proteins (Proteinortho6 with default parameters), see Supplementary Table S1. It is not surprising, given that the same sequences are aligned just with differing starting points. Although *st*(*S*_*n*_, *S*_*m*_) ≠ *st*(*S*_*m*_, *S*_*n*_) in general, the reciprocal bitscores for any two proteins of these sets are highly similar. With that, one can assume *st*(*S*_*n*_, *S*_*m*_) ∼ *st*(*S*_*m*_, *S*_*n*_), hence the calculation of *st*(*S*_*m*_, *S*_*n*_) can be omitted by estimating the scores based on *st*(*S*_*n*_, *S*_*m*_) as described in the Method section. This reduces the algorithmic effort by a factor of two. E-values calculated in this way strongly correlate with the reciprocal E-values ( $R_{\text{adj}}^{2}$ = 0.99). This correlation between the pseudo and classic approach is stronger compared to any comparison between two homology search tools using the classic approach. For more details see Supplementary Data Sheet S1.

### 3.4 Adaptive clustering

Regular clustering of a CC is performed by bisecting it into two sub-CCs of maximized connectivity until a predefined algebraic connectivity threshold is met. The default threshold applied by Proteinortho was defined empirically. Instead of working with a fixed threshold, the adaptive clustering strategy (core) assumes that members of orthologous groups should be found in all species. Iterative spectral clustering is applied until the component would split into two subcomponents of which neither covers all species that the original CC covered. The algorithm is aimed to keep orthologous groups as big as they need to be to cover all initially present species, even if the connectivity criterion is not met yet. This strategy is meant to identify the pan-genome as, e.g., the basis for reconstructing phylogenetic supertrees based on the reconstruction of trees from multiple orthologs.

Table 2 shows an overview of the number of reported orthologous groups relative to the percentage of species covered in the dataset. We found a high number of core-groups, i.e., orthology groups that span all input species, using the adaptive clustering, especially compared to the default connectivity threshold for the QfO dataset 2020/04. A comparable number of core-groups is found with a very low threshold of 1*e* ^−5^ but at the same time increases the maximal number of proteins per group dramatically. Overall the core module shows the best trade-off between the number of core-groups and size. It is worth noting that the results of the core approach differ drastically from the results from Proteinortho5 (ARI: 0.35).

**TABLE 2:** Key performance indicators of different clustering parameters applied to the QfO benchmark dataset 2020/04 (78 species). Similarity: ARI compared to the Proteinortho5 clustering with default parameters, classic: classic adaptive reciprocal best hit algorithm, *: default, *α*: algebraic connectivity threshold, core: adaptive clustering as described in 2.3.4, pseudo: pseudo-reciprocal sequence comparison strategy as described in 2.2.

	ortho-groups					Core-groups		
	Total	0%–25% species	25%–50% species	50%–75% species	75%–100% species	Total	max(proteins/group)	Similarity
Proteinortho5								
default (BLAST)	84 k	80 k	3 k	988	97	0	0	1
default clustering (DIAMOND)	79 k	75 k	3 k	984	97	0	0	.816
Proteinortho6 with DIAMOND sensitive								
pseudo *α* = 0.1*	72 k	67 k	3 k	1 k	105	0	0	.822
classic *α* = 0.1	72 k	68 k	3 k	1 k	106	1	97	.821
classic *α* = 0.05	65 k	61 k	3 k	1 k	147	1	97	.819
classic *α* = 0.2	79 k	75 k	3 k	994	60	0	0	.797
classic *α* = 0.3	83 k	79 k	3 k	831	40	0	0	.769
classic *α* = 0.01	53 k	48 k	3 k	1 k	231	9	149	.706
classic *α* = 0.5	90 k	87 k	2 k	484	11	0	0	.692
classic *α* = 0.75	99 k	97 k	2 k	186	10	0	0	.606
classic + core	44 k	40 k	2 k	1 k	392	51	706	.352
classic *α* = 0.005	48 k	43 k	3 k	1 k	262	12	152	.15
classic *α* = 0.001	42 k	37 k	3 k	1 k	319	30	315	.141
classic *α* = 0.00001	36 k	32 k	2 k	1 k	377	50	3 k	.0923

### 3.5 Scalability

Proteinortho6 implements a number of upgrades that improve the processing time to a level that matches recent tools for the identification of orthologs such as SonicParanoid2 (Cosentino and Iwasaki, 2023) without compromising the quality of the predictions. As large-scale orthology assessment relies on pairwise sequence comparisons, processing time grows quadratically with the number of proteins to be compared. This number correlates with the number of species in an orthology analysis. To portray the scalability and thus the processing time relative to the size of analyses, we used a real-world dataset. It is based on randomly sampled proteomes of the bacteria kingdom provided by UniProt (UniProt-Consortium, 2018). Details can be found in the 1.4 section.

**FIGURE 1:**
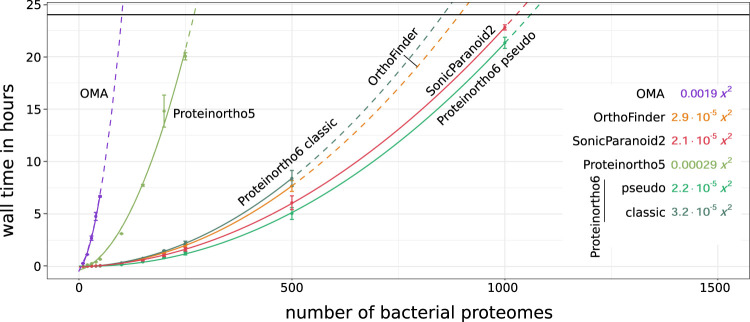
Scalability of total orthology prediction, including the all-versus-all sequence comparison and clustering, relative to dataset size of randomly selected bacterial proteomes of UniProt 2022_03 (Bac_10,20, … ,1000_). Average processing times are indicated by circles and fitted using a quadratic function (solid line, $R_{\mathrm{adj}}^{2}\geq 0.99$ ) for extrapolation (dashed lines). Classic: classic adaptive reciprocal best hit algorithm, pseudo: pseudo-reciprocal sequence comparison strategy as described in 2.2. Details on parameters and versions can be found in the Supplementary Data Sheet S1 and Supplementary Table S1.

Figure 1 shows the processing time and Supplementary Data Sheet S1 the memory consumption for an orthology analysis as a function of the number of species. OMA and Proteinortho5 exhibited the poorest scaling in terms of processing time, with a quadratic coefficient of 1.9 ⋅ 10^–3^ and 2.9 ⋅ 10^–4^ respectively, making an application to large species sets unfavorable. OrthoFinder, SonicParanoid2 and Proteinortho6 scale significantly better with the number of species. Proteinortho6 applying the classic reciprocal best alignment heuristic scales similarly to OrthoFinder in terms of processing time and outperforms the alternatives in terms of memory consumption. SonicParanoid2 and the pseudo reciprocal best alignment heuristic of Proteinortho6 show the best overall scaling results in regards to both metrics.

### 3.6 QfO sensitivity bias

An assessment of orthology prediction quality can be performed using Quest for Orthologs (QfO). The evaluation tool offers various tests to measure the precision and recall of predictions from different perspectives (for more information, refer to the Materials section). As exemplified below, we noticed a bias in the evaluation tool regarding the recall metric. True orthology relations can only be estimated based on existing data, e.g., via shared GO terms or congruence to curated species trees or datasets (Altenhoff and Dessimoz, 2009). Some QfO tests use the number of predicted orthologs as a proxy for sensitivity or recall, which translates into the number of edges in the orthology graph. In turn, the metric prefers large graphs. We exemplify this based on the results of “OrthoMCL” (Hickman, 2021) and “SonicParanoid_sensitive” (Consentino, 2021), which are among the highest recall scores across the different benchmarks. The referenced results of “SonicParanoid_sensitive” from 78 species include an orthology group that comprises over 407,000 proteins that are predicted to be co-orthologous to one another. Similarly, the results of “OrthoMCL” contain a group with 3,249 proteins. The biological informativeness of such a large group, in particular in relation to the small number of input species is questionable at best. In comparison, the largest group Proteinortho reports contain 3.5 proteins per species (with default clustering). In our observations, there appears to be a consistent trend where an increased count of edges generally results in higher sensitivity or recall scores across most benchmarks.

**FIGURE 2:**
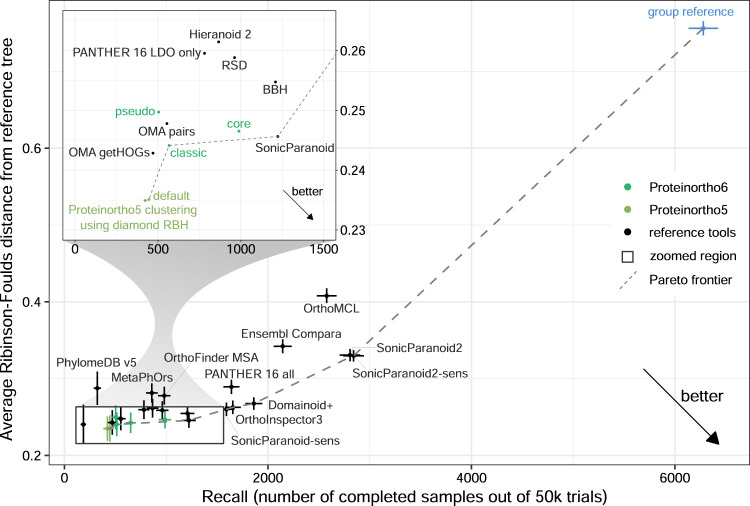
Assessment of Proteinortho and selected orthology tools provided by the QfO benchmark service using the 2020/04 dataset. The Generalized Species Tree Discordance benchmark Luca (G-STD2-Luca) with zoomed region. Proteinortho provides high precision at the cost of recall when used with default settings and slight variations between the different variants (classic: classic adaptive reciprocal best hit algorithm, pseudo: pseudo-reciprocal sequence comparison strategy as described in 2.2, core: adaptive clustering as described in 2.3.4, Proteinortho5 with BLAST and DIAMOND). The blue outlier on the right was generated using Proteinortho6 with DIAMOND and a relaxed clustering step (*α* = 0.00001, group reference).

To further exemplify this bias we constructed a “group reference” using Proteinortho6 with DIAMOND and a relaxed clustering that results in huge reported groups (*α* = 0.00001). To magnify the effect, we opted to work with groups instead of a list of pairs, where every pair of proteins within a group was predicted to be orthologous. In total “group reference” contained approximately ten to twenty times as many orthologs as most other tools like “SonicParanoid” or Proteinortho5. This approach achieved a Pareto optimal solution with high recall, as shown in Figure 2 e.g., for a Generalized Species Tree Discordance benchmark. Similar effects could be observed for almost all benchmark results (see Supplementary Data Sheet S1 and Supplementary Table S1). We are questioning this metric used and the strength of the Pareto optimal solution as a benchmark system as it is tied to this metric. Consequently, we are primarily evaluating the precision measurements of the benchmarks, as they remain unaffected by this bias.

### 3.7 QfO assessment

We found that using Proteinortho6 classic, which utilizes the classic adaptive best hit algorithm, with default clustering (ssyevr) mostly achieves precision scores within the top 25% and otherwise among the top 50% for all benchmark tests with the exception of the VGNC benchmark as summarized in Table 3. In general, all Proteinortho parameterizations and variations produce below-average precision scores in the VGNC benchmark. Proteinortho6, including the pseudo extension, showed similar precision scores to those obtained with Proteinortho5, with the majority of the benchmarks ranking within the top 25%. Overall, precision scores are similar to the Proteinortho5 results with mean log_2_ ratios below 0.005. Exchanging BLAST with DIAMOND in Proteinortho5 results in similar but slightly improved scores. Regarding Proteinortho parameterizations, the adaptive clustering (core) performs slightly worse overall with an improvement of −0.028.

**TABLE 3:** Quantifying Orthology Inference Precision: Assessing Proteinortho and Other Tools Using precision metrics of QfO benchmark dataset 2020/04. Three categories of benchmarks were employed: phylogeny-based benchmarks, function-based benchmarks, and reference orthology-based benchmarks, see the Method section for more details. A full description of all tools and the detailed benchmark results can be found in Supplementary Table S1. Proteinortho parameters are given in the form X + Y, where X specifies variation in the reciprocal best hit algorithm and Y the clustering modus. improvement: average log_2_ improvement relative to Proteinortho5 default. classic: classic adaptive reciprocal best hit algorithm. *: new default configuration of Proteinortho6, pseudo: pseudo-reciprocal sequence comparison strategy as described in 2.2, core: adaptive clustering as described in 2.3.4, flooding: flooding heuristic as described in 2.3.2. Group reference: Proteinortho6 with DIAMOND and a relaxed clustering step (*α* = 0.00001). ∇: RBH output of Proteinortho6 using DIAMOND in sensitive mode used for clustering with Proteinortho5. 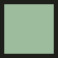: top 25%, 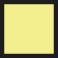: top 50% of published tools.

Benchmark type	Functional		Phlyogeny							Reference				
Metric	Avg Schlicker		Avg. Robinson-foulds							PPV				
Benchmark	EC	GO	GSTD2 Eukaryota	GSTD2 Fungi	GSTD2 Luca	GSTD2 Vertebrata	STD bacteria	STD Eukaryota	STD Fungi	SwissTrees	TreeFam-A	VGNC	# Top 25%	improvement
Proteinortho5														
classic + default													10	0
DIAMOND RBH^∇^ + default													10	0.02
Proteinortho6 with DIAMOND sensitive														
classic + default													10	−0.001
pseudo + default *													10	0.003
classic + core													8	−0.028
classic without clustering													9	−0.014
classic + flooding													9	−0.017
group reference													0	−1.473
published tools														
Domainoid+													0	−0.082
Ensembl Compara													0	−0.272
Hieranoid 2													9	−0.028
MetaPhOrs v.2.5													2	−0.135
OMA GETHOGs													4	−0.059
OMA Pairs													7	−0.007
OrthoFinder MSA v2.5.2													1	−0.125
OrthoInspector 3													1	−0.056
OrthoMCL													0	−0.648
PANTHER 16 al l													0	−0.183
phylomedb v5													4	−0.099
RSD													4	−0.107
RBH/BBH													6	−0.052
SonicParanoid													8	−0.032
SonicParanoid-fast													8	−0.015
SonicParanoid-mostsensitive													2	−0.059
SonicParanoid-sens													5	−0.043
SonicParanoid2													0	−0.224
SonicParanoid2-sens													0	−0.228

We found that the flooding heuristic performed similarly to the case without any clustering, highlighting the validity of this approach as a fallback system for the clustering if the size of a CC extends the capabilities of the spectral clustering algorithm. The conceptually simplified versions pseudo mode exhibited slightly better precision scores that are very similar to the results of Proteinortho5.

The orthology prediction results of “OMA Pairs”, “SonicParanoid”, and “SonicParanoid-fast” showed high precision specific to the phylogenetic benchmarks, where at least 6/7 benchmarks are among the top 25%. The largest average differences were found in comparison to “OrthoMCL” (−0.648 improvement), “Ensembl Compara” (−0.272 improvement) and “SonicParanoid2” (−0.224 improvement). Additionally, “OMA Pairs” produces the overall closest results compared to Proteinortho. A full assessment of all benchmarks can be found in the Supplementary Table S1.

**FIGURE 3:**
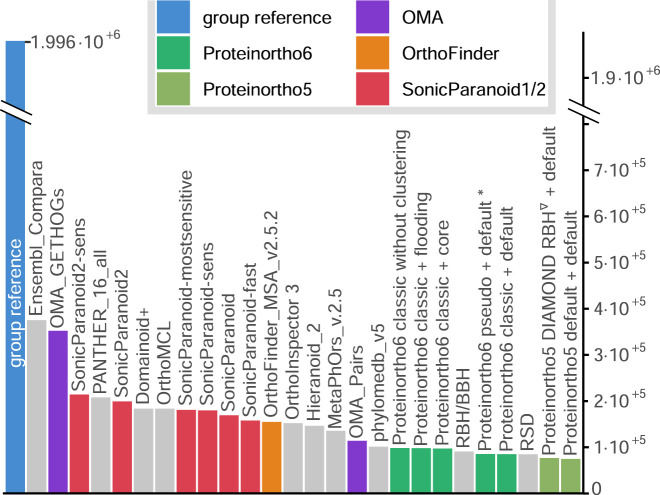
Number of ortholog relations in the function-based GO benchmark. Proteinortho parameters are given in the form X + Y, where X specifies variation in the reciprocal best hit algorithm and Y the clustering modus. classic: classic adaptive reciprocal best hit algorithm. *: new default configuration of Proteinortho6. pseudo: pseudo-reciprocal sequence comparison strategy as described in 2.2. core: adaptive clustering as described in 2.3.4. flooding: flooding heuristic as described in 2.3.2. Group reference: Proteinortho6 with DIAMOND using DIAMOND in sensitive mode. Names correspond to the tool names provided by QfO.

In the context of sensitivity scores, Proteinortho consistently yields some of the lowest scores, as demonstrated in detail in Supplementary Data Sheet S1. For example, the number of ortholog relations in the function-based Gene Ontology (GO) benchmark, is depicted in Figure 3. Proteinortho6 generates approximately 10 k orthologs, comparable to that reported by Proteinortho5 and “OMA pairs”. In contrast, “SonicParanoid” generates around 20 k orthologs, while the highest sensitivity scores are achieved by “Ensembl Compara” and “OMA GETHOGs,” which report between 30 k and 40 k orthologs.

### 3.8 Usability

Proteinotho6 is now readily available across various operating systems through multiple repositories, namely, Bioconda (Conda), Homebrew (Brew), and the Debian apt repository. Additionally, a containerized version of Docker can be obtained from quay. io. Proteinotho6 is now actively developed on GitLab fostering collaborative development and providing a transparent platform for community involvement. Furthermore, we implemented continuous integration and continuous deployment (CI/CD) routines through GitLab, ensuring efficient and seamless updates and frequent releases.

In order to assist researchers with limited programming experience, a graphical interface has been developed, which facilitates the generation of command lines and allows for the exploration of the output related to the orthology groups. Moreover, Proteinotho6 is now accessible in usegalaxy.eu (tools-iuc), providing a graphical interface and free computing resources for users. For large datasets, Proteinortho6 now includes a convenient interface to deploy jobs to multiple computing nodes in an HPC (High-Performance Computing) environment for Slurm systems. Furthermore, the clustering algorithm of Proteinotho6 is now not limited to Proteinotho output formats and now can be used on any undirected graph in the widespread ABC format.

Proteinortho6 was implemented with a focus on minimizing dependencies to ensure portability and avoid conflicts between multiple installed programs (“dependency hell”). In the Bioconda repository, Proteinotho6 has only 10 direct dependencies, while similar programs such as SonicParanoid2 and OrthoFinder have 15 and 14 dependencies, respectively.

## 4 Discussion

Proteinortho was designed to predict (co-)ortholog groups, with a focus on large datasets. Previous implementations have been unable to keep up with the deluge of newly sequenced genomes that calls for the analysis of millions of proteins and pairwise best-match graphs with billions of edges. With Proteinortho6, we present a comprehensive algorithmic update for both, the similarity comparisons, and the clustering step.

Based on the detailed evaluation, the sensitive variation of DIAMOND replaces BLAST in the sequence comparison step. This leads to a considerable speedup with an acceptable loss of sensitivity in the initial reciprocal best-hit graph. Proteinortho6 offers the use of all similarity search tools listed above as an alternative. An example is ucsc BLAT. It offers an even higher speedup at the cost of sensitivity. It primarily reports very similar sequences. This might be desirable if the dataset comprises only closely related species. We further explored an improved search strategy for the reciprocal best hit calculations, the pseudo approach. Results proved similar to classic strategies while consistently yielding an additional significant speed up. To optimize the performance, the pseudo option has been selected as the new default *modus operandi*. This method has the potential for broader adoption in other tools in the field.

In the clustering procedure of Proteinortho6, a new strategy is implemented to compute the algebraic connectivity and the associated Fiedler vector using the Fortran library Lapack, which is significantly faster for connected components of larger sizes. The analysis of real-world connected components in combination with artificially generated ones shows the superiority of Lapack’s ssyevr approach over the original power iteration in terms of running time. The precision evaluation showed no major changes. A downside, however, is the quadratic memory requirement of ssyevr. Very large connected components are inevitable when analyzing large datasets. Technically these would be workable through the power iteration. However, at the enormous cost of CPU time. Hence, the flooding heuristic was introduced. The reworked clustering implementation also makes efficient use of multiple CPU cores and can even be distributed among multiple computing nodes.

A regular application of orthology tools is the calculation of robust phylogenetic reconstructions via a supertree analysis based on single-copy orthologs among a given set of species. The new adaptive clustering facilitates better results in this context as it automatically optimizes the clustering parameters for each group to cover as many species as possible without overestimating the amount of paralogs. Besides this specific research question, core falls behind the default clustering approach in terms of precision and thus is not chosen as the default.

For the comparison with other orthology prediction tools and databases, the standardized QfO benchmark system was used. Despite the usefulness of the benchmark system, we encountered some shortcomings that may affect the comparisons. In particular, the recall metric of the system is biased towards large inputs. Execution parameters and tool versions are typically not documented. Nevertheless, the precision estimates provided by QfO gave valuable insights regarding changes in the quality of our predictions when introducing alternative algorithms. Results generated by Proteinortho are consistently among the highest-performing tools in terms of precision and archived scores are generally close to the results of OMA. In terms of sensitivity, Proteinortho produces among the lowest scores compared to the other tools, highlighting a distinct trade-off. Proteinortho6 notably excels in terms of execution time and provides a considerable speedup over its previous implementation. This substantially increases the size of datasets that can be processed and makes efficient use of the hardware provided.

## Data Availability

Publicly available datasets were analyzed in this study. This data can be found here: ftp://ftp.ebi.ac.uk/pub/databases/reference_proteomes/previous_releases/qfo_release-2020_04_with_updated_UP000008143/QfO_release_2020_04_with_updated_UP000008143.tar.gz (QfO Release 2020_04 = 2020.2), https://ftp.uniprot.org/pub/databases/uniprot/previous_major_releases/ release-2022_Proteinortho6 is published under GNU GPLv3 license. It is available via http://www.bioinf.uni-leipzig.de/Software/proteinortho/ Release 2022_03).

## Supplementary material

Supplementary Table S1 Additional figures and results Cluster Algorithm Overview, Scalability, Table 1 with alternative datasets, Sensitivity Assessment, Small World Phenomenon Example, QfO Evaluation All Plots, E-value Comparison.

Supplementary Data Sheet S1 Listings, including datasets, proteome identifier, execution times, and raw datasets overview : sizes of all datasets used, scalability uniprot n : raw data of Figure 1, clustering evaluation : raw data of Table 2, sensitivity, precision of BLAST alternatives : raw data of Tab. 1, qfo 2020_04 benchmark results : raw data of Table 3, power iteration vs lapack for 1 thread : raw data of the analysis in Section 2.2, shuffled Bac_n_ 2022_03 : proteome identifier of the Bac_n_ dataset, BigCC dataset : proteome identifier of the BigCC dataset, QfO release 2020_04 with updated UP000008143 dataset : proteome identifier of the QfO 2020/04 dataset.

## Abbreviations

l_2_FC, log_2_ fold-change; RBH, reciprocal best hit graph; RF, Robinson-Foulds; GSTD, generalized species tree discordance test; PPV, positive predictive value; H, hours; GB, gigabyte; ARI, adjusted rand index; k, thousand; QfO, Quest for Orthologs.
