# Supplementary material for: Proteinortho6: pseudo-reciprocal best alignment heuristic for graph-based detection of (co-)orthologs
Source: Front Bioinform. 2023 Dec 13;3:1322477. doi: 10.3389/fbinf.2023.1322477 (PMC10751348; doi:10.3389/fbinf.2023.1322477)
Supplement: Supplementary file 1 [file DataSheet1.PDF]

Supplementary Material

1 CLUSTER ALGORITHM OVERVIEW

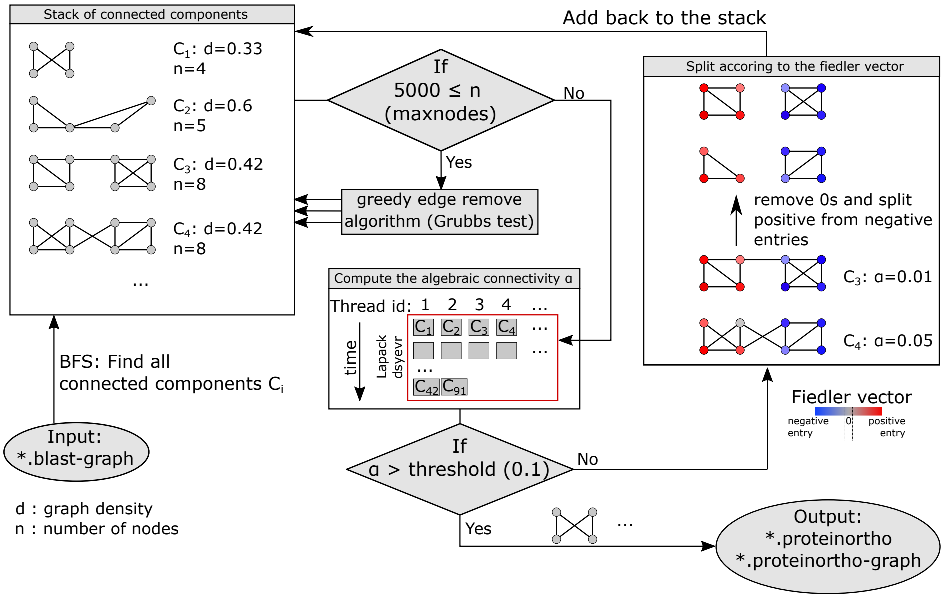

Figure S1: Updated multi-threading system for the clustering step. First, all connected components are identified in the input graph using the branch-first search algorithm (BFS). Suitable small components are processed in parallel using `Lapack ssyevr`. The remaining larger components are processed using the greedy split algorithm. Resulting components with insufficient algebraic connectivity are split according to the associated Fiedler vector and marked for an additional round of processing.

2 SCALABILITY

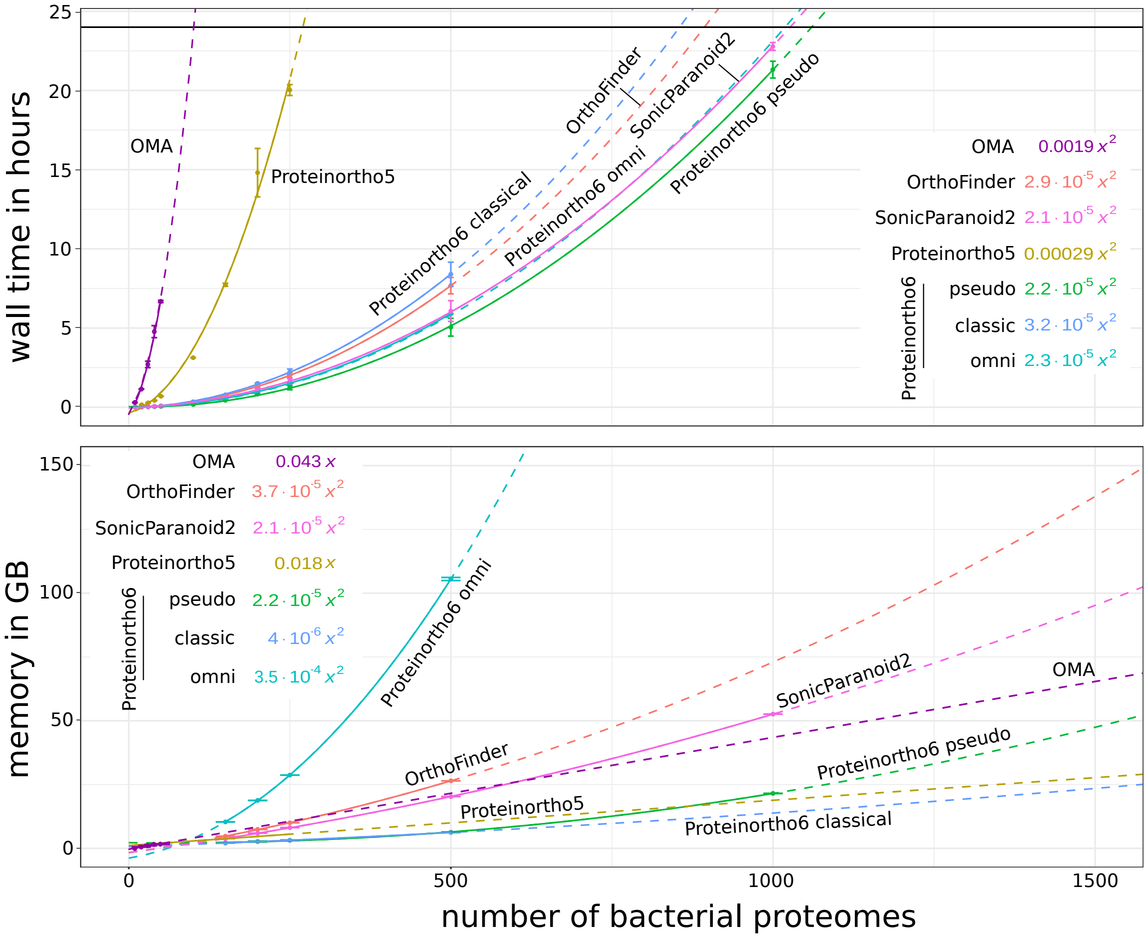

Figure S2: Scalability of total orthology prediction, including the all-versus-all sequence comparison and clustering, relative to dataset size of randomly selected bacterial proteomes of UniProt 2022_03 ($\mathtt{Bac}_{10,20,...,1000}$). Average processing times and peak memory consumption are indicated by circles and fitted using a quadratic function (solid line, $R^2_{adj} \geq 0.99$ for wall time and $R^2_{adj} \geq 0.89$ for memory consumption) for extrapolation (dashed lines). The peak memory consumption was restricted to a dataset of size ≥ 150. Because of a negative quadratic term, the memory consumption of `Proteinortho5` and `OMA` was fitted using a linear function instead. Coefficients of the term with the highest degree are indicated for each tool. Details on parameters and versions can be found in the Supplemental Table

3 TAB. 1 ALTERNATIVE DATASETS

3.1 `EFD`

`EFD` is a dataset of 29 food-related and probiotic strains of the Lactobacillus genus Bonacina et al. (2017). It represents a small set of very similar species: Enterococcus durans IPLA655 RAST, Enterococcus faecalis 19116 RAST, Enterococcus faecalis 2924 RAST, Enterococcus faecalis MB5259 RAST, Enterococcus faecalis PC1.1 RAST, Enterococcus faecalis str. Symbioflor 1 RAST, Enterococcus faecium CRL1879 RAST, Enterococcus faecium E1604 RAST, Enterococcus faecium E1613 RAST, Enterococcus faecium L-3 RAST, Enterococcus faecium L-X RAST, Enterococcus faecium NRRLB-2354 RAST, Enterococcus

faecium T110 RAST, Enterococcus faecium UC10237 RAST, Enterococcus faecium UC7251 RAST, Enterococcus faecium UC7256 RAST, Enterococcus faecium UC7267 RAST, Enterococcus faecium UC8668 RAST, Enterococcus faecium UC8733 RAST, Enterococcus hirae INFE1 RAST, Enterococcus malodoratus ATCC43197 RAST, Enterococcus mundtii ATCC882 RAST, Enterococcus mundtii CRL1656 RAST, Enterococcus mundtii CRL35 RAST, Enterococcus raffinosus cftri2200 RAST, Lactobacillus johnsonii NCC 533 RAST, Lactococcus garvieae Lg2 RAST, Lactococcus lactis subsp. cremoris MG1363 RAST, Listeria monocytogenes HCC23 RAST.

Table S1. Tab. 1 with the `EFD` dataset. Sensitivity and precision are given relative to the `BLAST` results in line 1. Edges: number of edges in the initial orthology graph; wall time: total processing time; memory: peak memory usage; l_2FC: $\log_2$ fold change relative to `Proteinortho5` results; $*$: default option of `Proteinortho6`. Ranks are indicated: (green) top 25%, (yellow) top 50%.

algorithm	edges	sensitivity %	precision %	wall time l_2FC *sec*	memory l_2FC GB
`Proteinortho5.16b`	713482	100	100	0<br>476.67	0<br>1.13
`ucscblat`	485383	67.7	99.51	4.6<br>19.77	2.7<br>0.17
`diamond`	682860	94.7	98.94	3.8<br>34.93	2.5<br>0.2
`diamond sensitive`	708887	98.36	98.99	2.3<br>99.55	2.3<br>0.23
`diamond sensitive pseudo`	708438	98.21	98.91	3.2<br>52.9	2.5<br>0.2
`diamond ultrasens`	711673	98.72	98.97	0.83<br>267.38	2.2<br>0.25
`diamond fast`	645098	89.51	99	4.1<br>28.03	2.7<br>0.18
`lastp`	689038	95.58	98.97	4.2<br>26.51	2.4<br>0.22
`lastp m100`	697589	96.85	99.05	2.7<br>73.56	2.1<br>0.26
`lastp m1000`	699126	97.08	99.08	-0.16<br>531.89	2.1<br>0.26
`mmseqsp`	701887	97.5	99.11	1<br>236.58	0.27<br>0.94
`mmseqsp s1`	635394	88.27	99.12	2.1<br>107.69	0.3<br>0.92
`mmseqsp s7.5`	706721	98.15	99.08	-0.51<br>680.31	0.25<br>0.95
`rapsearch`	649000	89.98	98.92	2<br>116.87	0.43<br>0.84
`topaz`	695431	96.42	98.92	1.7<br>147.52	2.2<br>0.24
`topaz fast`	695431	96.42	98.92	1.7<br>146.82	2.2<br>0.25
`ublast`	706013	97.48	98.51	3.4<br>45.8	2.2<br>0.25
`usearch`	661522	91.01	98.16	3.7<br>36.74	2.7<br>0.17

3.2 Bac$_n$

The Bac dataset comprised all bacterial reference proteomes from UniProt, release 2022/03 (UniProt-Consortium, 2018). This set was downsampled to incremental subsets of random proteomes. For instance, Bac$_{10}$ contains 10 randomly selected bacterial proteomes, Bac$_{20}$ extends this set by 10 additionally randomly selected proteomes, and so on. A full list is shown in the Supplemental Table.

Table S2. Tab. 1 with the Bac$_{20}$ dataset. Sensitivity and precision are given relative to the BLAST results in line 1. Edges: number of edges in the initial orthology graph; wall time: total processing time; memory: peak memory usage; l$_2$FC: log$_2$ fold change relative to Proteinortho5 results; $*$: default option of Proteinortho6. Ranks are indicated: [green] top 25%, [yellow] top 50%.

algorithm	edges	sensitivity %	precision %	wall time l$_2$FC *min*	memory l$_2$FC GB
Proteinortho5.16b	158904	100	100	0<br>9.46	0<br>1.2
ucscblat	9577	5.9	97.92	5.4<br>0.23	3.2<br>0.13
diamond	118073	67.7	91.12	5.2<br>0.26	2.7<br>0.19
diamond sensitive	154046	89.83	92.67	3.7<br>0.75	2.2<br>0.27
diamond sensitive pseudo	154807	89.8	92.18	4.6<br>0.39	2.4<br>0.22
diamond ultrasens	159059	92.48	92.39	2.3<br>1.98	2<br>0.31
diamond fast	80123	45.6	90.44	5.5<br>0.21	3<br>0.15
lastp	131498	77.1	93.17	5.4<br>0.22	2.5<br>0.21
lastp m100	139055	82.07	93.79	3.6<br>0.79	2.1<br>0.28
lastp m1000	141621	83.66	93.87	0.46<br>6.9	2<br>0.29
mmseqsp	137330	81.48	94.28	2<br>2.33	0.37<br>0.93
mmseqsp s1	71262	41.65	92.87	3.6<br>0.78	0.38<br>0.92
mmseqsp s7.5	142732	84.65	94.25	0.22<br>8.1	0.31<br>0.97
rapsearch	67064	39.08	92.61	3.2<br>1.02	-0.036<br>1.23
topaz	132274	77.51	93.11	2.6<br>1.51	2<br>0.29
topaz fast	132274	77.51	93.11	2.7<br>1.46	2<br>0.3
ublast	140835	79.86	90.11	4.6<br>0.39	2.2<br>0.26
usearch	108073	59.56	87.58	4.5<br>0.43	3<br>0.15

Table S3. Tab. 1 with the $\texttt{Bac}_{50}$ dataset. topaz did not finish (core dump). Sensitivity and precision are given relative to the BLAST results in line 1. Edges: number of edges in the initial orthology graph; wall time: total processing time; memory: peak memory usage; l_2FC: $\log_2$ fold change relative to Proteinortho5 results; $*$: default option of Proteinortho6. Ranks are indicated: [green] top 25%, [yellow] top 50%.

algorithm	edges	sensitivity %	precision %	wall time l_2FC h	memory l_2FC GB
Proteinortho5.16b	1076306	100	100	0<br>0.67	0<br>2.22
ucscblat	81270	7.4	98.08	5.1<br>0.02	3.8<br>0.16
diamond	814578	69.15	91.37	5.1<br>0.02	3.1<br>0.26
diamond sensitive	1046328	90.11	92.7	3.3<br>0.07	2.5<br>0.39
diamond sensitive pseudo	1050090	89.98	92.22	4.5<br>0.03	2.9<br>0.3
diamond ultrasens	1078707	92.63	92.43	1.7<br>0.21	2.4<br>0.41
diamond fast	567748	47.9	90.82	6.1<br>0.01	3.5<br>0.2
lastp	900483	77.91	93.13	5.1<br>0.02	2.9<br>0.3
lastp m100	949104	82.65	93.72	3.1<br>0.08	2.5<br>0.4
lastp m1000	966106	84.21	93.82	-0.12<br>0.73	2.4<br>0.43
mmseqsp	942364	82.4	94.12	1.5<br>0.24	1.2<br>0.95
mmseqsp s1	511480	44.27	93.17	3.1<br>0.08	1.3<br>0.93
mmseqsp s7.5	977547	85.4	94.03	-0.33<br>0.84	1.2<br>0.98
rapsearch	481648	41.59	92.94	2.7<br>0.1	0.33<br>1.76
ublast	962830	80.75	90.26	4.5<br>0.03	2.6<br>0.37
usearch	722121	59.12	88.12	4.1<br>0.04	3.6<br>0.18

Table S4. Tab. 1 with the $\texttt{Bac}_{200}$ dataset. `topaz` did not finish (core dump). Sensitivity and precision are given relative to the `BLAST` results in line 1. Edges: number of edges in the initial orthology graph; wall time: total processing time; memory: peak memory usage; l_2FC: $\log_2$ fold change relative to `Proteinortho5` results; $*$: default option of `Proteinortho6`. Ranks are indicated: [green] top 25%, [yellow] top 50%.

algorithm	edges	sensitivity %	precision %	wall time l_2FC h	memory l_2FC GB
`Proteinortho5.16b`	18786311	100	100	0<br>12.73	0<br>4.78
`ucscblat`	1379336	7.18	97.9	5.2<br>0.35	4.5<br>0.21
`diamond`	14109018	68.27	90.9	4.9<br>0.44	3.6<br>0.4
`diamond sensitive`	18218261	89.79	92.59	3.3<br>1.28	3<br>0.58
`diamond sensitive pseudo`	18275224	89.64	92.15	4.3<br>0.64	3.4<br>0.45
`diamond ultrasens`	18798520	92.41	92.35	1.8<br>3.58	2.9<br>0.65
`diamond fast`	9786172	46.96	90.16	5.5<br>0.29	4<br>0.29
`lastp`	15595713	77.08	92.85	4.8<br>0.46	3.3<br>0.47
`lastp m100`	16473700	82.07	93.59	3.1<br>1.49	2.8<br>0.7
`lastp m1000`	16783613	83.73	93.72	-0.029<br>12.99	2.6<br>0.77
`mmseqsp`	16371099	81.91	93.99	1.6<br>4.26	2.3<br>0.96
`mmseqsp s1`	8835896	43.62	92.74	3.3<br>1.33	2.3<br>0.94
`mmseqsp s7.5`	17009354	85.05	93.94	-0.2<br>14.6	2.2<br>1.03
`rapsearch`	8373338	41.14	92.31	2.8<br>1.84	1.3<br>1.97
`ublast`	16773637	80.29	89.92	4.1<br>0.75	2.9<br>0.66
`usearch`	12648992	58.84	87.39	4.2<br>0.68	4.3<br>0.25

4 SENSITIVITY ASSESSMENT

Table S5. Quantifying Orthology Inference Sensitivity: Assessing Proteinortho and Other Tools Using sensitivity metrics of QfO benchmark dataset 2020/04. Three categories of benchmarks were employed: phylogeny-based benchmarks, function-based benchmarks, and reference orthology-based benchmarks, see the Method section for more details. A full description of all tools and the detailed benchmark results can be found in Supplemental Table. `improvement`: average $\log_2$ improvement relative to `Proteinortho5 default+default`. `Proteinortho` parameters are given in the form X+Y, where X specifies variation in the reciprocal best hit algorithm and Y the clustering modus. `classic`: classic adaptive reciprocal best hit algorithm. $*$: new default configuration of `Proteinortho6`. group reference: `Proteinortho6` with `DIAMOND` and a relaxed clustering step ($\alpha = 0.00001$). ∇: RBH output of `Proteinortho6` using `DIAMOND` in `sensitive` mode. TPR: true positive rate. num: number of orthologs. (green): top 25%, (yellow): top 50% of published tools.

benchmark type	functional		phlyogeny							reference				
metric	num		completed tree samples							TPR				
benchmark	EC	GO	GSTD2 Eukaryota	GSTD2 Fungi	GSTD2 Luca	GSTD2 Vertebrata	STD Bacteria	STD Eukaryota	STD Fungi	SwissTrees	TreeFam-A	VGNC	# top 25%	`improvement`
`Proteinortho5`:														
`default` + `default`													0	0
`DIAMOND` RBH$^{\nabla}$ + `default`													0	0.035
`Proteinortho6` with `DIAMOND` `sensitive`:														
`default` + `default`													0	0.251
`classic` + `core`													0	0.481
`pseudo` + `default` $*$													0	0.246
`classic` without clustering													0	0.495
`classic` + `flooding`													0	0.482
group reference													10	2.047
published tools:														
Domainoid+													5	1.013
Ensembl Compara													5	1.172
Hieranoid 2													0	0.724
MetaPhOrs v.2.5													3	0.791
OMA GETHOGs													2	0.618
OMA Pairs													0	0.415
OrthoFinder MSA v2.5.2													6	0.959
OrthoInspector 3													0	0.944
OrthoMCL													9	1.172
PANTHER 16 all													8	1.066
phylomedb v5													0	0.446
RSD													0	0.524
RBH/BBH													1	0.634
SonicParanoid													0	0.804
SonicParanoid-fast													0	0.632
SonicParanoid-mostsensitive													0	0.975
SonicParanoid-sens													0	0.938
SonicParanoid2													10	1.176
SonicParanoid2-sens													11	1.207

5 SMALL WORLD PHENOMENON

With rising numbers of species, the connected components tend to expand quickly, leading to the formation of extensive connected components. `Proteinortho` v6.3.0 with default parameters using `diamond` (v2.0.15) but without the clustering step was used to process randomly selected bacterial proteomes $\texttt{Bac}_n$ until a size of $n = 1000$ species and the `BigCC` dataset with 1800 species. From the output, the largest connected component is determined and put in relation to the total number of nodes in the graph. The resulting growth is illustrated in Fig. S3.

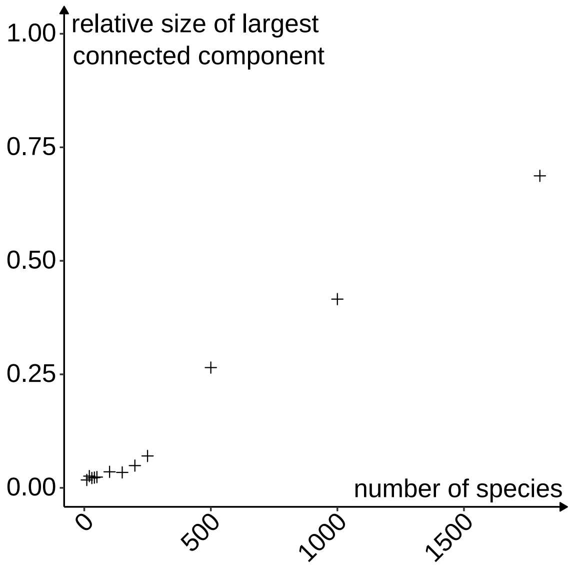

Figure S3: The size of the largest connected components relative to the total number of nodes from randomly selected bacterial proteomes of `UniProt` until a size of 1000 and the `BigCC` dataset with 1800 species. The graphs were built using `Proteinortho` with default parameters.

6 QFO EVALUATION

The following plots show all QfO benchmark results of the 2020_{20} dataset (2020.2) using the following configurations of `Proteinortho`:

1. default_step2_po5: `Proteinortho5` with default settings
2. po5_clustering_using_diamond: `Proteinortho5` with default clustering with an input graph that was generated using `Proteinortho6` with `diamond` with default parameters.
3. omni_bin1k_step2_diamond : `Proteinortho6` in omni modus using bin=1k (bin size) and diamond in sensitive modus.
4. pseudo_step2_diamond : `Proteinortho6` in pseudo modus using diamond in sensitive modus.
5. conn0.1_diamond : `Proteinortho6` in canonical modus (canonical reciprocal best hit algorithm) using diamond in sensitive modus.
6. core_diamond_coreMaxProts10 : `Proteinortho6` in canonical modus (canonical reciprocal best hit algorithm) using diamond in sensitive modus and the clustering modus core with the parameter coreMaxProts=10 (maximal number of proteins of groups per species)

6.1 Phylogeny-Based Definition Benchmarks

6.1.1 Species Tree Discordance Benchmark

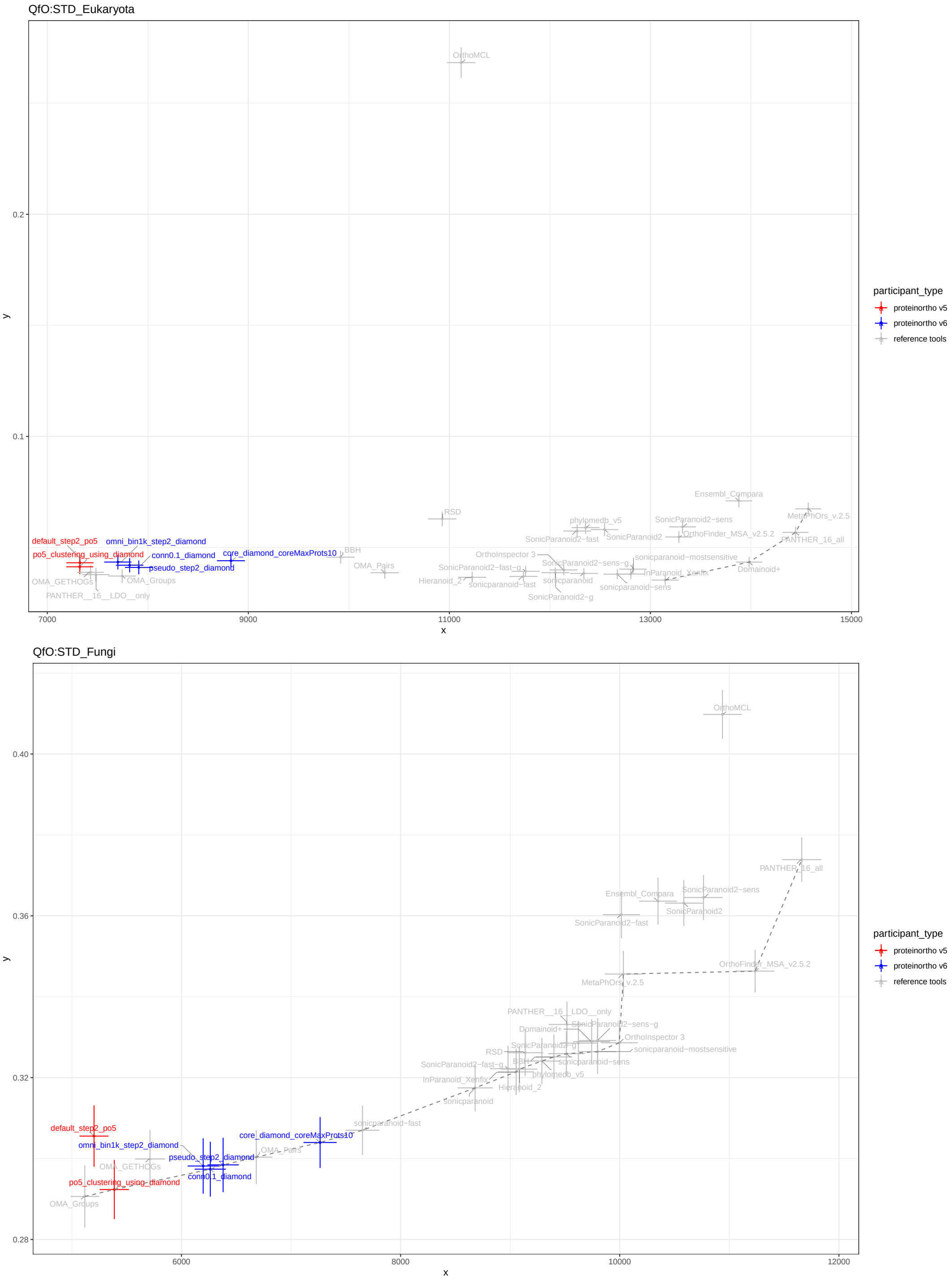

Figure S4: Species Tree Discordance Benchmark 1/2. x: Recall - completed tree samples, y: Avg. Robinson-Foulds distance

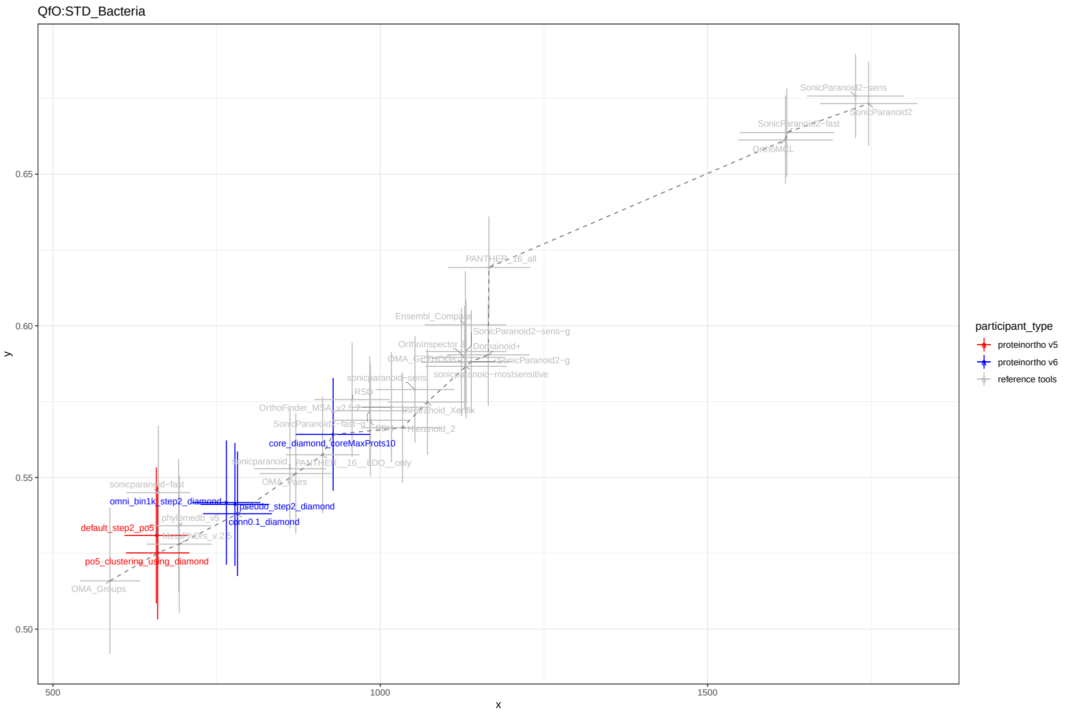

Figure S5: Species Tree Discordance Benchmark 2/2. x: Recall - completed tree samples, y: Avg. Robinson-Foulds distance

6.1.2 Generalized Species Tree Discordance Benchmark

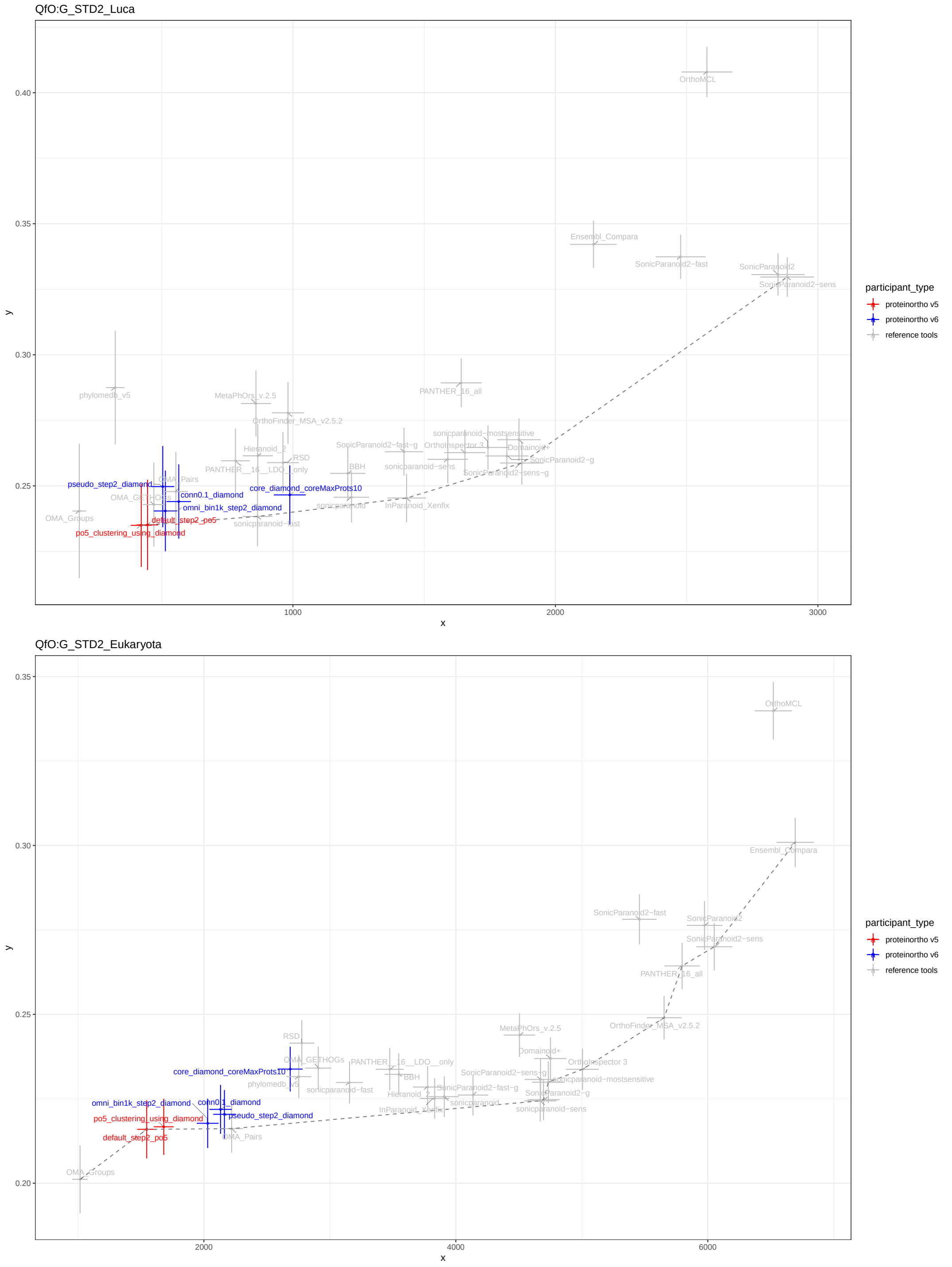

Figure S6: Generalized Species Tree Discordance Benchmark 1/2. x: Recall - completed tree samples, y: Avg. Robinson-Foulds distance

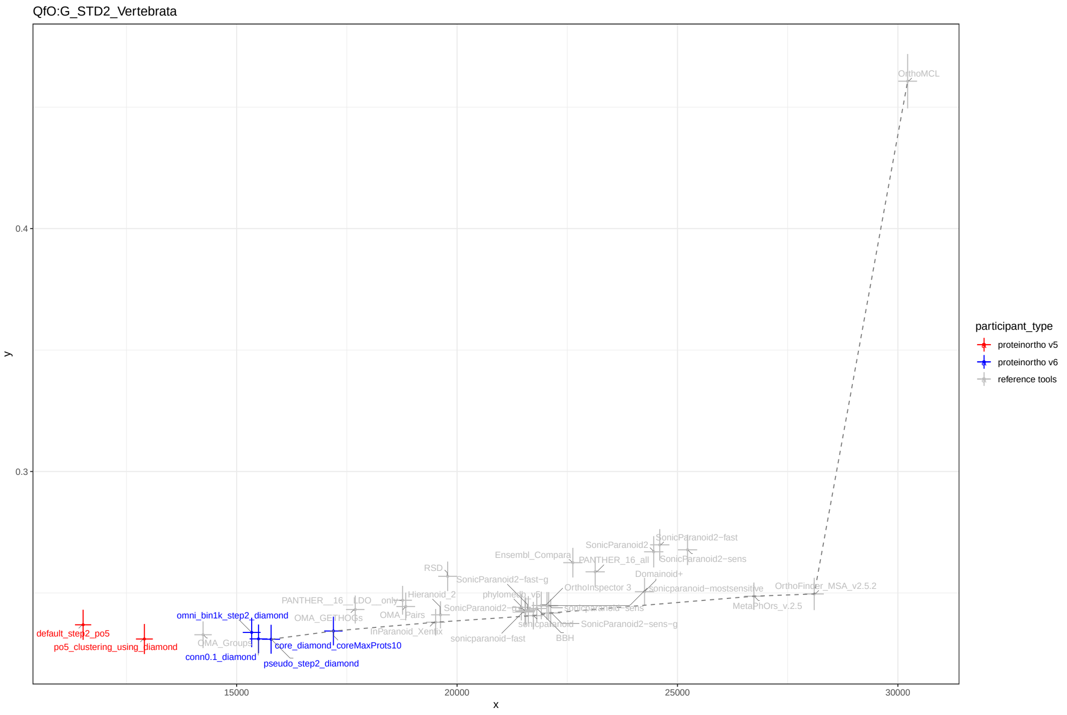

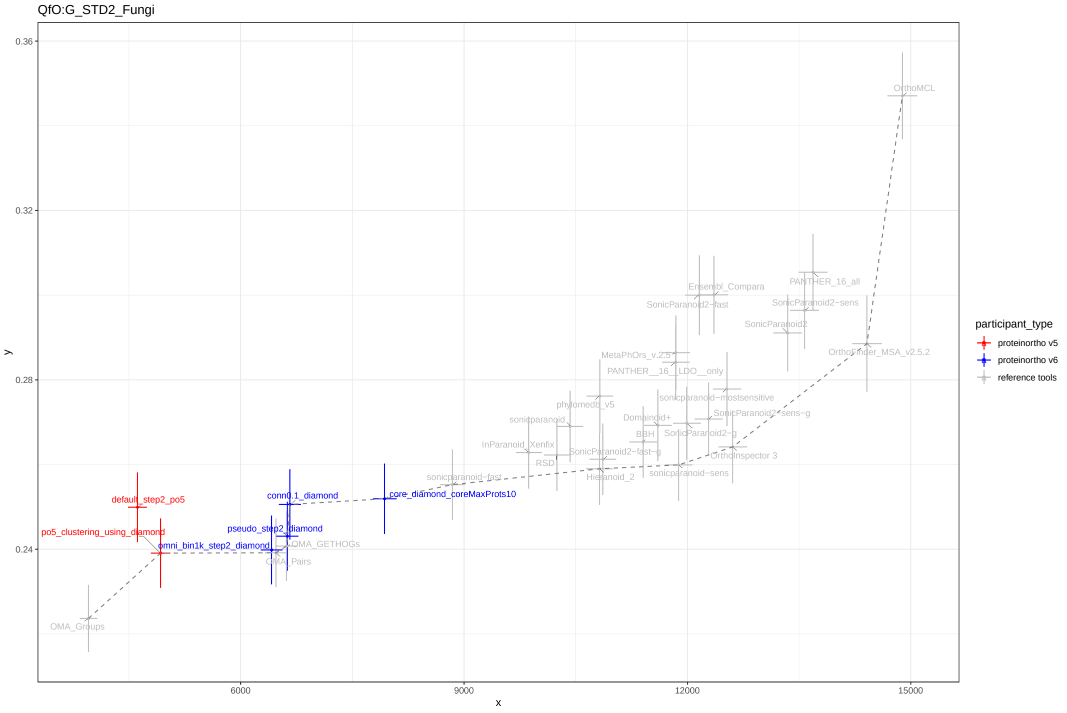

Figure S7: Generalized Species Tree Discordance Benchmark 2/2. x: Recall - completed tree samples, y: Avg. Robinson-Foulds distance

6.2 Reference Orthology Based Benchmarks

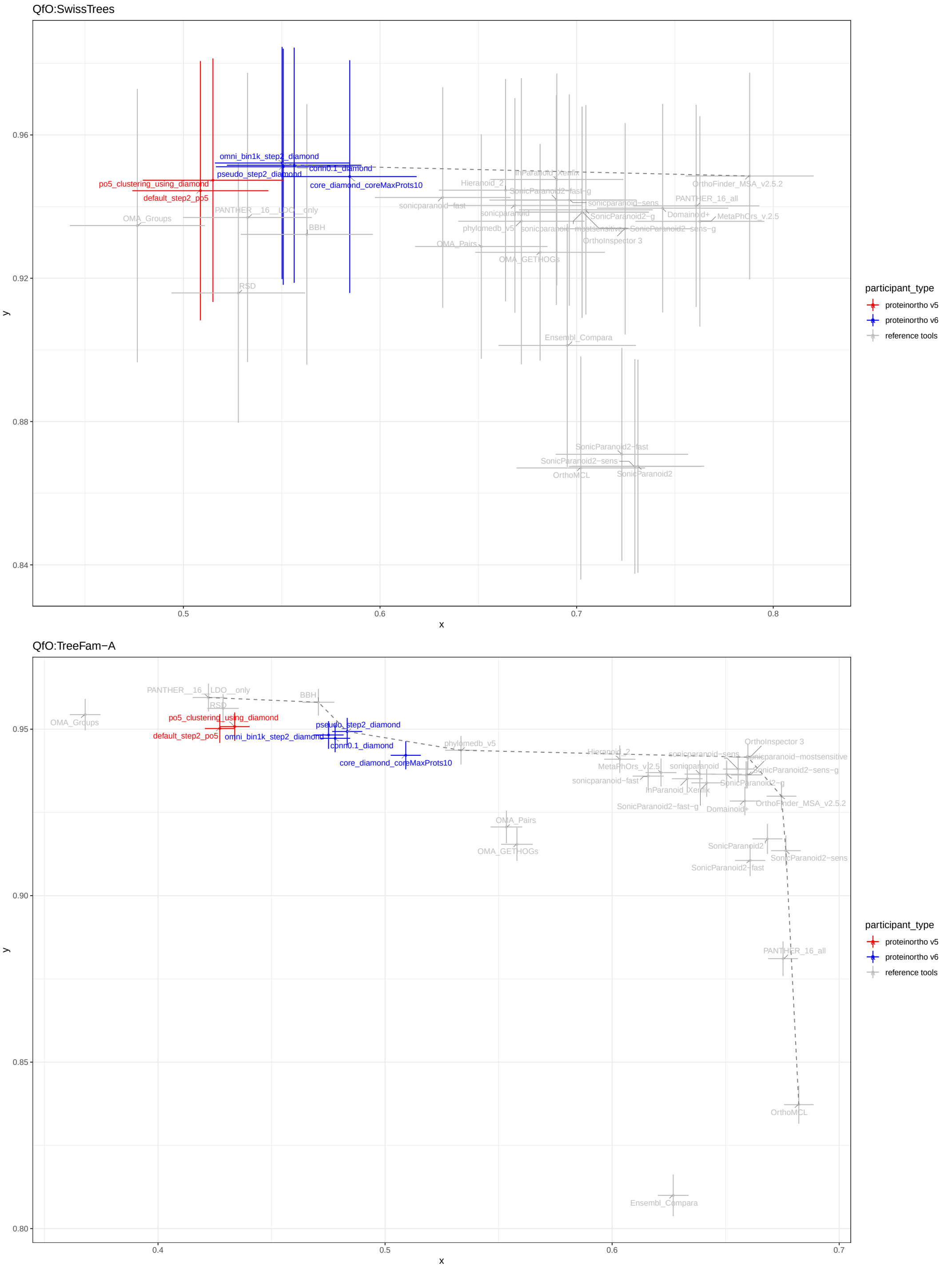

Figure S8: Reference Orthology Based Benchmarks 1/2. x: True Positive Rate (TPR), y: Precision / Positive Predictive Value (PPV)

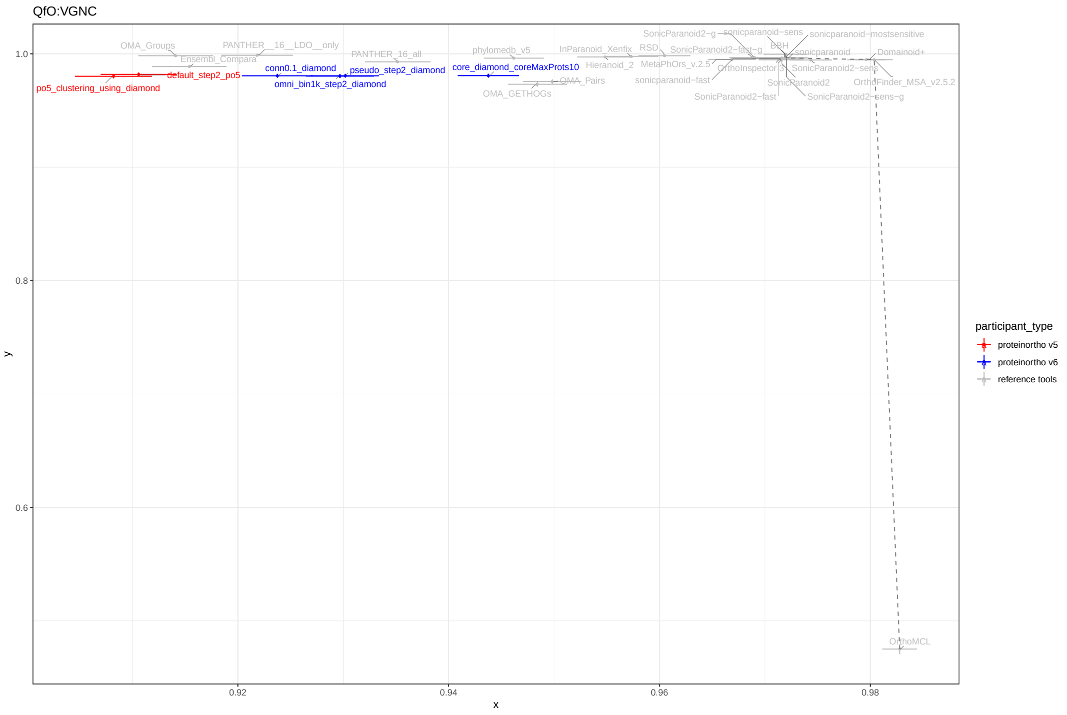

Figure S9: Reference Orthology Based Benchmarks 2/2. x: True Positive Rate (TPR), y: Precision / Positive Predictive Value (PPV)

6.3 Function-Based Benchmarks

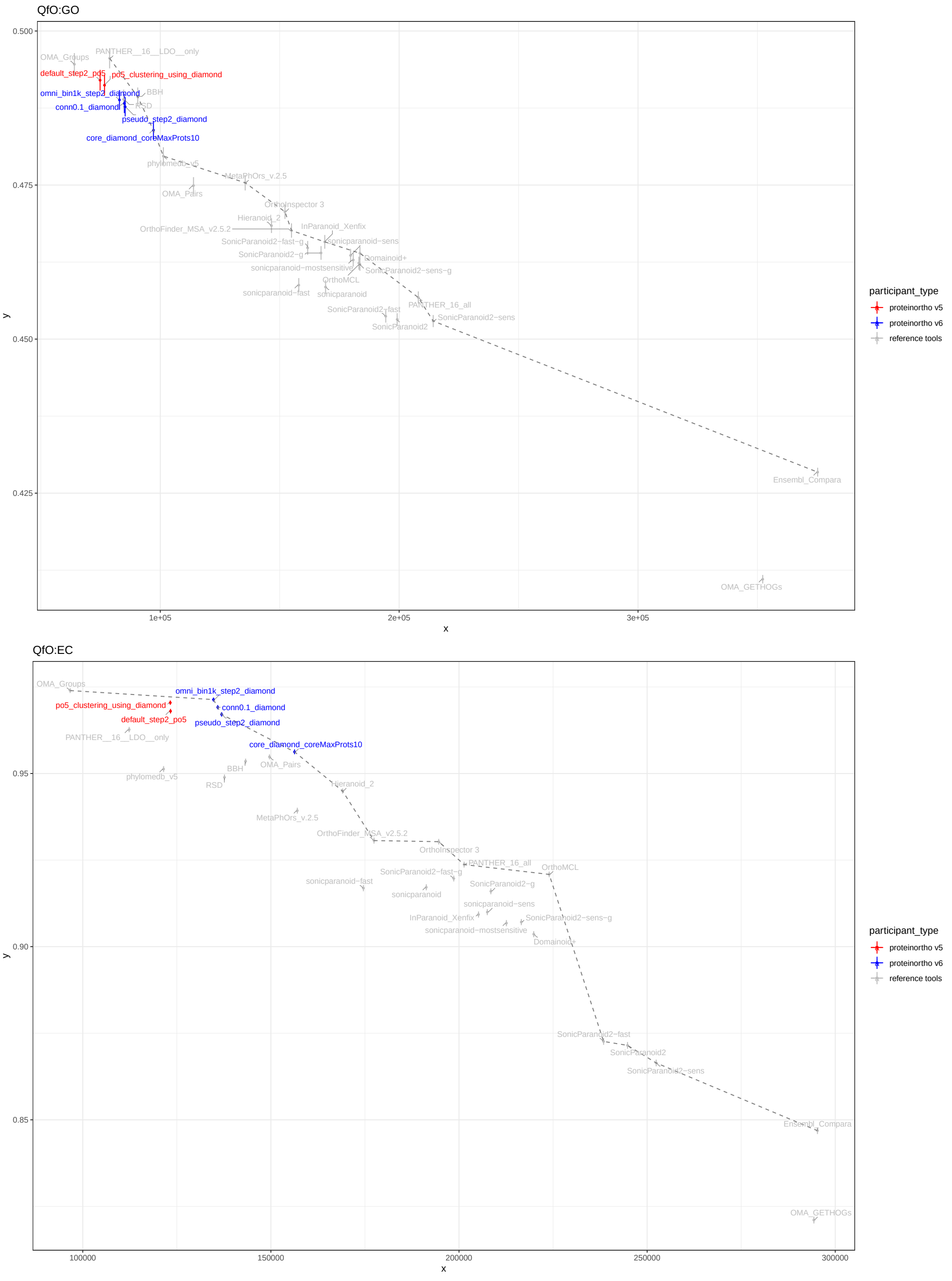

Figure S10: Function-Based Benchmarks. x: Recall - Number of Ortholog Relations, y: Precision - Avg. Schlicker Similarity

7 E-VALUE LINEAR REGRESSION ANALYSIS

Linear regression analysis of between different homology search programs. For two algorithms X and Y, (for example `BLAST` and `diamond`) first a classical reciprocal best hit graph is built for each program using `Proteinortho6` without clustering. The resulting BLAST graphs are then compared using R, such that for each protein pair that is found in both graphs (called ”match” in the plots) all combinations between the reported E-values are collected and correlated.

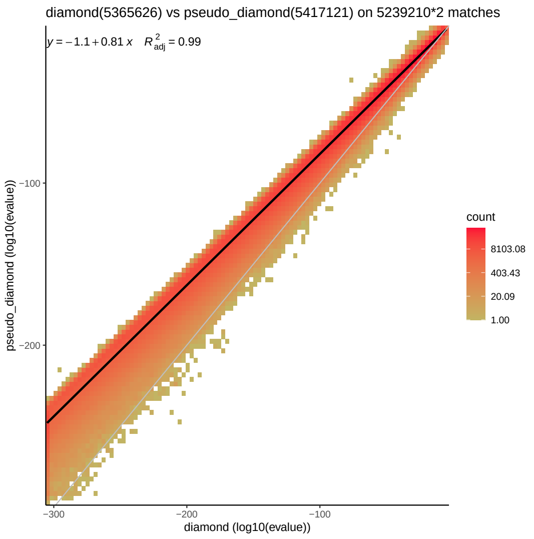

(m) diamond vs pseudo diamond

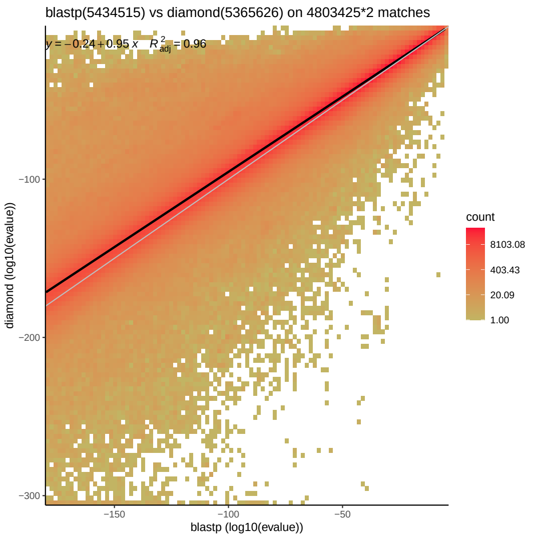

(n) BLAST vs diamond

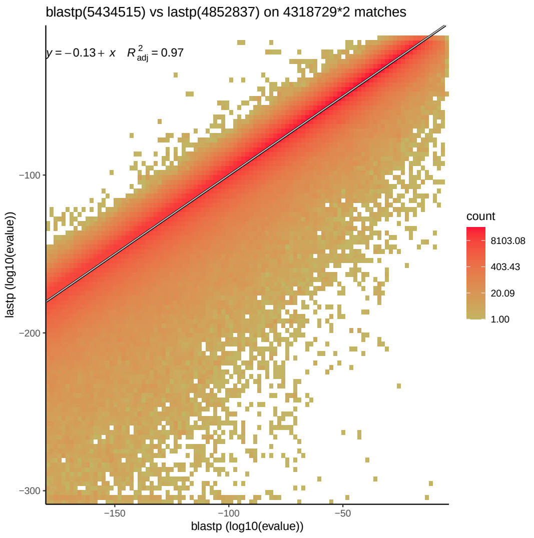

(o) BLAST vs last

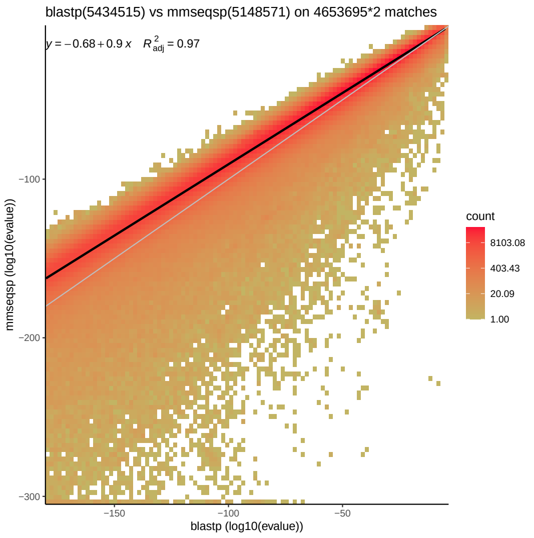

(p) BLAST vs MMSeqs2

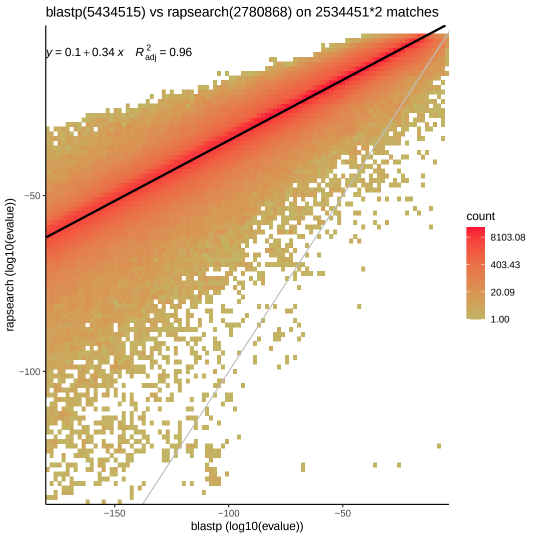

(q) BLAST vs RAPSearch2

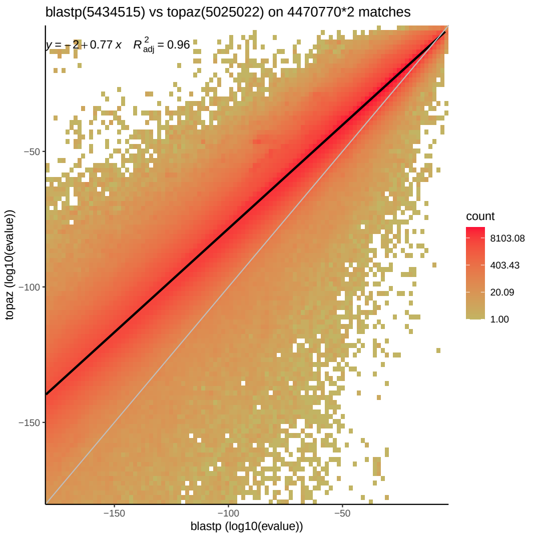

(r) BLAST vs topaz

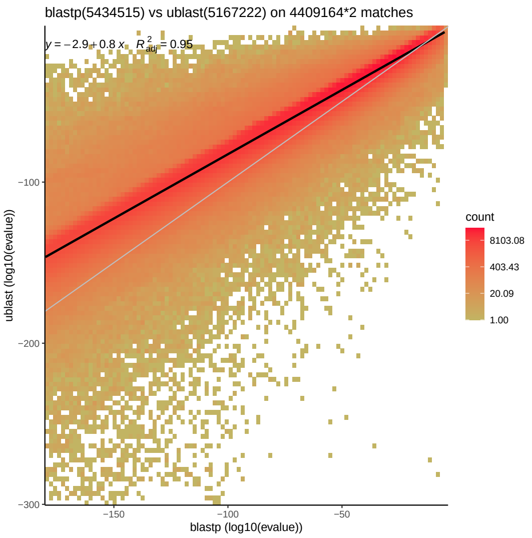

(s) BLAST vs ublast

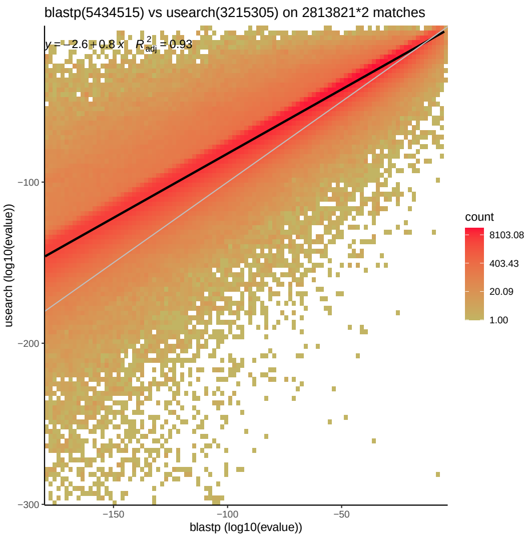

(t) BLAST vs usearch

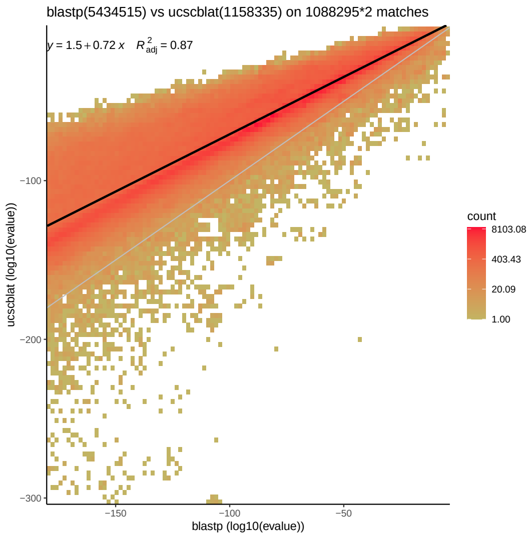

(u) BLAST vs blat

Figure S12: Linear regression analysis of log10 transformed E-values of `pseudo` transformed values and E-values using the canonical reciprocal best hit algorithm of `Proteinortho6`. The gray line indicates the identity function y=x. diamond: diamond in sensitive mode

REFERENCES

Bonacina, J., Suárez, N., Hormigo, R., Fadda, S., Lechner, M., and Saavedra, L. (2017). A genomic view of food-related and probiotic Enterococcus strains. *DNA research* 24, 11–24. doi:10.1093/dnares/dsw043

UniProt-Consortium (2018). Uniprot: a worldwide hub of protein knowledge. *Nucleic acids research* 47, D506–D515
